# Carbon doping switching on the hydrogen adsorption activity of NiO for hydrogen evolution reaction

**DOI:** 10.1038/s41467-020-14462-2

**Published:** 2020-01-30

**Authors:** Tianyi Kou, Mingpeng Chen, Feng Wu, Tyler J. Smart, Shanwen Wang, Yishang Wu, Ying Zhang, Shengtong Li, Supriya Lall, Zhonghua Zhang, Yi-Sheng Liu, Jinghua Guo, Gongming Wang, Yuan Ping, Yat Li

**Affiliations:** 1 Department of Chemistry and Biochemistry, University of California, 1156 High Street, Santa Cruz, CA 95064 USA; 2 Department of Physics, University of California, 1156 High Street, Santa Cruz, CA 95064 USA; 3 Department of Chemistry, University of Science and Technology of China, Hefei, Anhui 230026 PR China; 4 Key Laboratory for Liquid-Solid Structural Evolution and Processing of Materials (Ministry of Education), School of Materials Science and Engineering, Shandong University, Jingshi Road 17923, Jinan, 250061 PR China; 5 Advanced Light Source, Lawrence Berkeley National Laboratory, Berkeley, CA 94720 USA

**Keywords:** Catalyst synthesis, Catalytic mechanisms, Electrocatalysis

## Abstract

Hydrogen evolution reaction (HER) is more sluggish in alkaline than in acidic media because of the additional energy required for water dissociation. Numerous catalysts, including NiO, that offer active sites for water dissociation have been extensively investigated. Yet, the overall HER performance of NiO is still limited by lacking favorable H adsorption sites. Here we show a strategy to activate NiO through carbon doping, which creates under-coordinated Ni sites favorable for H adsorption. DFT calculations reveal that carbon dopant decreases the energy barrier of Heyrovsky step from 1.17 eV to 0.81 eV, suggesting the carbon also serves as a hot-spot for the dissociation of water molecules in water-alkali HER. As a result, the carbon doped NiO catalyst achieves an ultralow overpotential of 27 mV at 10 mA cm^−2^, and a low Tafel slope of 36 mV dec^−1^, representing the best performance among the state-of-the-art NiO catalysts.

## Abstract (web summary)

While H_2_ evolution from water may serve as a renewable source of fuel, there are a limited number of catalysts that are stable and active in alkaline media. Here, authors find carbon doping of NiO to increase the number of favorable sites for H_2_ evolution and boost electrocatalytic performances.

## Introduction

Water electrolysis represents a sustainable and environmentally friendly method to generate hydrogen fuel. Since proton rich environment is favorable for hydrogen adsorption on catalyst surface, acidic medium is preferable for hydrogen evolution reaction (HER). However, the acidic condition prohibits the use of non-platinum group metals as catalysts. In addition, the corrosive acidic fog generated by the acidic electrolyte not only contaminates the produced hydrogen gas, but also causes severe chemical corrosion of electrolyzers^1,2^. These factors add significant cost for hydrogen generation and pose barriers for constructing large-scale electrolyzers. Alternatively, alkaline electrolytes with low vapor pressure and relatively mild chemical environment could avoid these issues. More importantly, non-platinum group metals such as Ni can be used as electrocatalyst/electrode for alkaline water electrolysis. A major challenge for alkaline water electrolysis is the requirement of an additional water dissociation step (i.e., the cleavage of the strong H–OH bond) for generating the essential H atom intermediates for HER. The high activation barrier of water dissociation makes HER very sluggish in alkaline medium^3^. For example, Pt typically exhibits two orders of magnitude lower exchange current density in alkaline solution than that in acidic solution^4^. It is therefore critical to develop alkaline HER catalysts that contain both hydrogen adsorption sites as well as water adsorption and dissociation sites^5,6^.

Transition metal oxides such as NiO are promising alkaline HER catalysts. Since Ni sites in NiO possess incompletely filled d orbitals, Ni sites was reported to serve as actives sites for water adsorption and dissociation in alkaline electrolyte^7,8^. For instance, Zhang et al.^9^ used NiO as a HER electrocatalyst that achieved an overpotential of 110 mV at the geometric current density (*j*_geo_) of 10 mA cm^−2^. Nonetheless, the performance of NiO is still not comparable to Pt-based catalysts. One of the possible reasons is lack of hydrogen adsorption sites^8^. A recent effort of integrating NiO with metallic Ni, which provides hydrogen adsorption sites, has further decreased the overpotential for alkaline HER to 80 mV at *j*_geo_ of 10 mA cm^−2^ (ref. ^6^). However, the susceptibility to oxidation of metallic Ni could affect the stability of the integrated catalyst. Moreover, given that only the Ni/NiO interface has the synergistic effect in alkaline HER, the integrated system may not be able to fully utilize the catalyst’s surface area.

Alternatively, we aimed to create hydrogen adsorption sites for NiO through heteroatom doping. Herein, we report a carbon doped Ni_1−x_O that shows an impressively low overpotential of 27 mV at *j*_geo_ of 10 mA cm^−2^ and a small Tafel slope of 36 mV dec^−1^ in KOH solution, which is comparable to the performance (14 mV at 10 mA cm^−2^, 29 mV dec^−1^) of the benchmark Pt/C catalyst. Structural analysis reveals that the carbon dopant substitutionally replaces a third-layer 6-coordinated Ni in NiO. DFT simulation further suggests that the carbon dopant distorts the local structure of NiO and decreases the coordination number of Ni. These under-coordinated Ni sites are highly favorable for hydrogen adsorption. In addition, the carbon sites serve as the hot spots for water dissociation with a fairly low energy barrier of 0.81 eV.

## Results

### The impact of carbon doping on NiO structural evolution

**Fig. 1:**
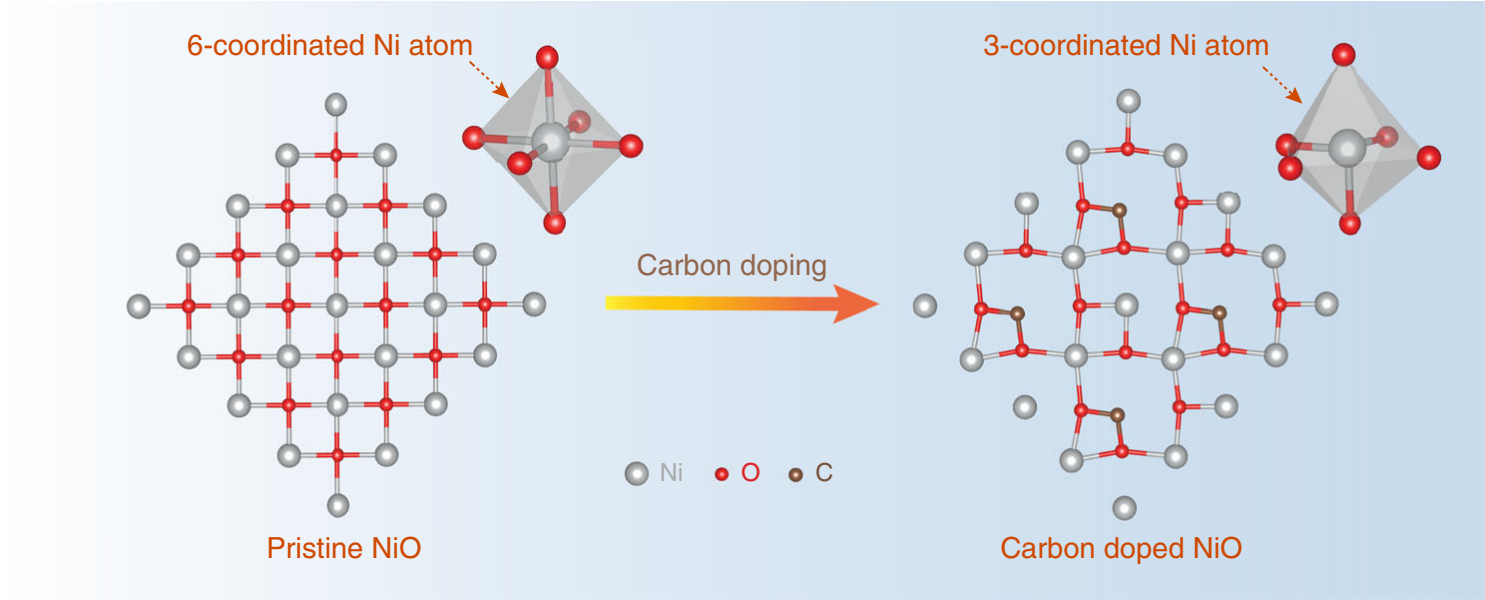
DFT calculated structure of pristine NiO and carbon doped NiO DFT calculations showing the carbon doping induced structure change of NiO (the color coding for different atoms is consistent for the whole paper).

Hydrogen adsorption property of a HER catalyst is largely determined by its surface electronic structure and coordination geometry. Introduction of heteroatom dopant can modulate the electron density and the coordination number of active sites, and consequently adjust their hydrogen adsorption behavior. Among different dopants, carbon is particularly attractive. In a previous report of C-doped TiO_2_, the addition of C dopant reduced the coordination number of Ti and increased the charge density of Ti (ref. ^10^). Accordingly, we first employed density functional theory (DFT) calculations to investigate the possible impact of carbon doping on the coordination geometry of NiO (Supporting Information). As shown in Fig. 1, Ni is 6-coordinated in pristine NiO, whereas the C doping causes the distortion of the NiO local structure because of the mismatch of the radius and coordination number between carbon and Ni. The distortion creates enough tensile strain on the Ni–O bond and subsequently cleaves the bond. As a result, it reduces the coordination number of Ni from 6 to 3, and thus increases the charge density of Ni, where the under-coordinated Ni could potentially act as active H adsorption sites in NiO. In addition, the high affinity of carbon to oxo groups could promote water adsorption or dissociation.

### Synthesis and structural characterization of C-doped NiO

**Fig. 2:**
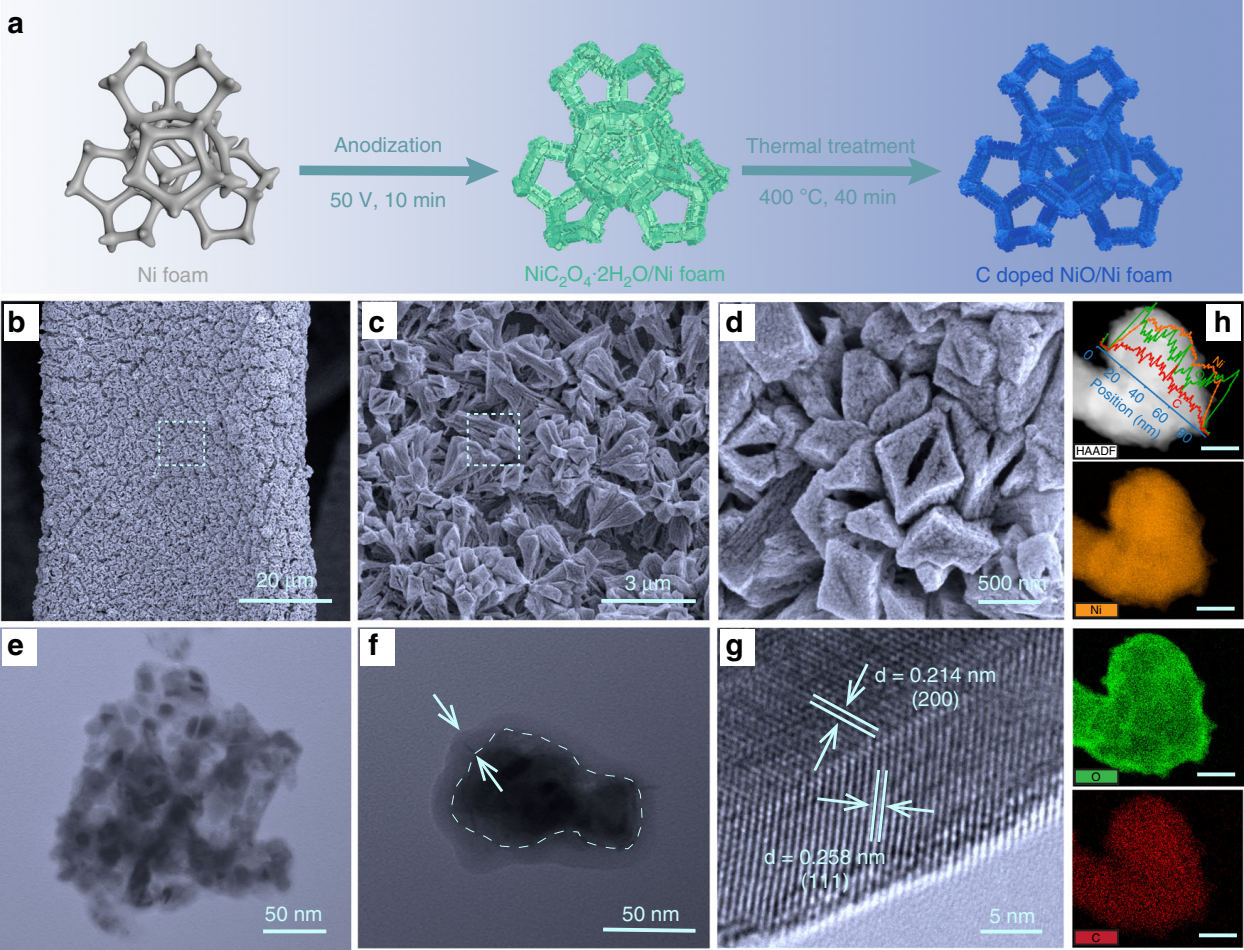
Preparation and structural characterization of C-doped NiO. **a** Schematic illustration of the synthesis of carbon doped NiO on NF. **b**–**d** SEM images of the C-doped NiO nanorod/NF in different magnifications. The regions highlighted by dashed boxes in (**b**) and (**c**) are magnified in (**c**) and (**d**), respectively. **e**–**f** TEM images of the fragments of C-doped NiO nanorod/NF. **g** A HR-TEM image collected at the edge of the nanoparticle in (**f**). **h** High-angle annular dark field (HAADF)-TEM image of a Ni core/NiO shell particle and the corresponding EELS elemental mapping of Ni, O, and C (scale bars are 20 nm).

Carbon doped NiO was prepared by a two-step process as illustrated in Fig. 2a. Nickel oxalate dihydrate (NiC_2_O_4_·2H_2_O) bulk crystals were first uniformly grown on a Ni foam (NF) via anodization at 50 V in oxalate acid as reported elsewhere^11^ (Supplementary Fig. 1). The NF coated with NiC_2_O_4_·2H_2_O crystals was then annealed in argon ambience at 400 °C, which is considerably higher than the decomposition temperature of NiC_2_O_4_·2H_2_O (Supplementary Fig. 2a). The decomposition changed the morphology of bulk crystals to porous rod structure (Fig. 2c, d). Transmission electron microscopy (TEM) images revealed that the rods are composed with small nanoparticle subunits (Fig. 2e). Notably, each nanoparticle is a core-shell structure (Fig. 2f). High resolution-TEM (HR-TEM) image collected from the edge of the nanoparticle showed lattice fringe spacings consistent with the *d*-spacings reported for (111) and (200) crystal planes of NiO (Fig. 2g), which is also in consistency with the NiO composition in X-ray diffraction (XRD) pattern (Supplementary Fig. 2b, and Supplementary Note 1), suggesting the shell is NiO. According to the XRD result, the core of the particle is metallic Ni. Electron energy loss spectroscopy (EELS) elemental mapping (Fig. 2h) and line scan (inset of Fig. 2h) were collected from a representative nanoparticle, which also confirms the copresence of Ni and O in the nanoparticle. The intense O signal obtained at the edge of the nanoparticle again supports the proposed Ni core-NiO shell structure. Significantly, a noticeable amount of carbon signal was also present over the entire nanoparticle, indicating the successful incorporation of carbon doping.

### Chemical environment of the carbon doped NiO

**Fig. 3:**
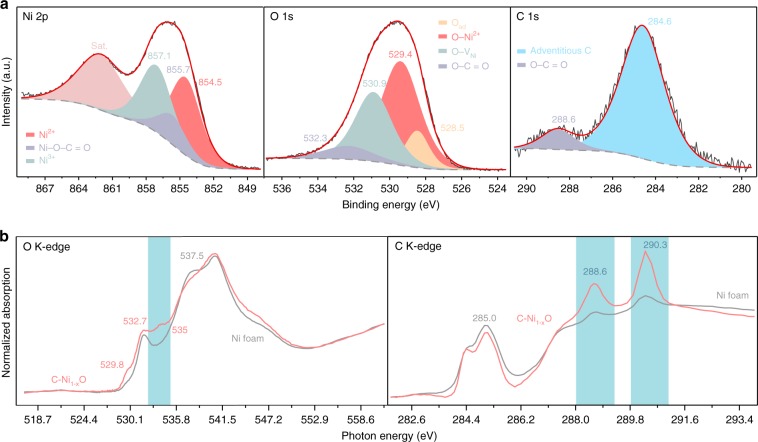
Chemical environment of C–Ni_1−x_O. **a** Ni 2p, O 1s, and C 1s XPS spectra collected from C–Ni_1−x_O particles. The black curves are the experimental data. The red curves are the summation of the deconvoluted peaks (shaded regions). **b** O K-edge and C K-edge XANES spectra of C–Ni_1−x_O grown on NF (red curve) and NF (gray curve).

X-ray photoelectron spectroscopy (XPS) measurements were performed to probe the chemical environment of each element in C-doped NiO (Fig. 3a). Ni 2p XPS spectrum exhibits two broad peaks centered at 862 (satellite peak) and 856 eV, respectively. The latter peak can be deconvoluted into three sub-peaks. The Ni 2p peak at 854.5 eV is consistent with the value reported for Ni^2+^ in NiO (ref. ^12^), while the peak centered at a higher binding energy of 857.1 eV corresponds to the signal of Ni^3+^ (refs. ^13,14^). It is noteworthy that we did not observe metallic Ni signal from the sample. The metallic Ni and Ni–C signals were only observed when the NiO shell was etched away by argon plasma, as evidenced by the peaks at 852.7 eV in the Ni 2p spectrum^15^ and 283.3 eV in the C 1s spectrum^16^ (Supplementary Fig. 3). The results are consistent with the EELS mapping and XRD results, and again confirm the Ni core/NiO shell structure. O 1s XPS spectrum also supports the presence of NiO. The deconvoluted peak located at 529.4 eV suggests O bond with Ni^2+^ (ref. ^14^). In addition, the peak located at 530.9 eV is assigned to the O adjacent to Ni vacancy^13^. The presence of Ni vacancy has been reported to result in valence increase of its vicinity Ni (Ni^2+^ to Ni^3+^) to achieve charge neutrality^13^, which is in consistency with the peak at 857.1 eV in Ni 2p spectrum^13^. C 1s spectrum shows two peaks centered at 284.6 and 288.6 eV, respectively. The former peak is due to the adventitious carbon^17^. The 288.6 eV signal suggests the presence of O–C=O bond, which is in consistent with the EELS results and agrees well with the observation of the O 1s peak of O–C=O at 532.8 eV (ref. ^10^). The O–C=O group creates a distinguishable coordination environment of Ni (Ni–O–C=O) in NiO, which leads to an additional signal (Ni–O–C=O) located at 855.7 eV in the Ni 2p spectrum. The atomic concentrations of Ni, O, and C (288.6 eV) were calculated to be 53.95%, 44.22% and 1.83%, respectively.

We also compared the X-ray absorption near edge spectroscopy (XANES) data of NF substrate and NF decorated with thermally treated NiC_2_O_4_·2H_2_O collected at O and C K-edge (Fig. 3b). The O K-edge XANES of the annealed NiC_2_O_4_·2H_2_O shows a prominent pre-peak at about 529.8 eV that corresponds to the transition between O 1s state and the hole state, which has been regarded as a characteristic signal of the Ni deficient NiO (i.e., Ni_1−x_O)^18,19^. The hybridization of the O 2p orbital with the Ni 4s orbital creates some unoccupied states for transitions in the NiO with energy around 537.5 eV. The presence of Ni vacancies reduces the intensity of 537.5 eV peak^19,20^. More importantly, absorption peaks between 532.7 and 535 eV (shaded region) were observed for the annealed NiC_2_O_4_·2H_2_O, which are due to the mixed transitions from O 1s to empty states of high valence Ni (Ni^3+^), and O 1s to the $\pi _{{\mathrm{C}} = {\mathrm{O}}}^ \ast$ (refs. ^20,21^). NF and thermally treated NiC_2_O_4_·2H_2_O have similar C K-edge spectra except for significantly different peak intensities at 288.6 and 290.3 eV. The carbon signals observed for NF substrate originates from adventitious carbon contamination^22^. However, the substantially higher peak intensities of the annealed NiC_2_O_4_·2H_2_O at 288.6 and 290.3 eV are unlikely related to adventitious carbon^23^. Instead, these peaks are attributed to the transition of C 1s to π* and σ* state in O–C=O (ref. ^24^). Taken together, the XPS and XANES results disclosed two important information. First, the NiO shell contains Ni vacancies and is Ni deficient. Second, carbon dopants are substitutionally replacing the Ni positions in Ni_1−x_O. Therefore, this NiC_2_O_4_·2H_2_O derived material is denoted as C–Ni_1−x_O, and our subsequent DFT simulation was performed based on this structural model.

### Modeling the surface and electronic structure of C–Ni_1−x_O

**Fig. 4:**
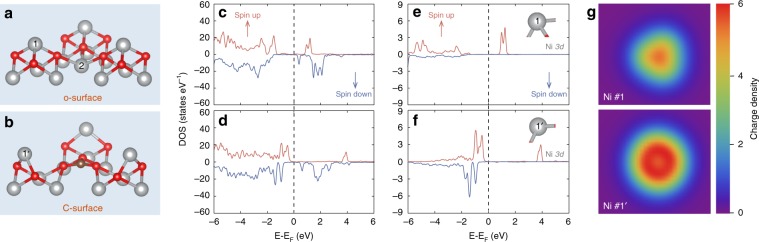
Surface structures, PDOS plots and Ni charge density mapping in o-surface and C-surface. **a**, **b** o-surface and C-surface structures with numbers labeling different Ni sites. **c**, **d** PDOS plots of the Ni 3d and O 2p orbitals of the first three layers from the o-surface and C-surface. **e**, **f** PDOS plots of the 3d orbitals of Ni on o-surface (Ni #1) and C-surface (Ni #1′), respectively. **g** Charge density mappings of the top-layer Ni (#1 and 1′) on o-surface (upper) and C-surface (bottom).

Since we observed (100) and (111) facets in the HR-TEM image collected from the Ni_1−x_O shell (Fig. 2g), both (100) (Supplementary Fig. 4) and (111) (Supplementary Fig. 5) surface models were built and relaxed for subsequent DFT simulation (Supplementary Table 1). (111) surface has two possible terminations: Ni termination and O termination. An investigation on the surface phase diagram of (111) facet shows that Ni termination is more stable than O termination in the Ni rich environment (Supplementary Fig. 6, and Supplementary Note 2). Combined with our realistic Ni rich synthesis condition, the (111) surface should also be terminated by Ni. In addition, (111) facet tends to have surface reconstruction and the reconstructed surface is thermodynamically more stable than the pristine Ni terminated (111) (Supplementary Fig. 6). The results are consistent with the previous report on the thermodynamic stability of NiO polar (111) surface^25^. Specifically, ¾ of the outermost ions and ¼ of the second outermost ions of the pristine (111) surface (denoted as p-surface) are missing during the surface reconstruction, resulting in a new surface exposed (i.e., octopolar surface, denoted as o-surface in Fig. 4a). Since there are more Ni ions missing compared with O ions in the surface reconstruction, Ni vacancies appear on the o-surface. As a result, high valence Ni^3+^ sites are generated to balance the charge^13^. The presence of Ni^3+^ sites is supported by XPS and XANES results. Furthermore, C substitutional doping was investigated for both (100) and o-surface. However, the only stable structure was obtained when C substitutes one third-layer 6-coordinated Ni (labeled as Ni #2 in Fig. 4a) in o-surface. Therefore, (100) surface is not considered in further discussion. Since the bond length of C–O bond (~1.4 Å) is much shorter than that of Ni–O bond (~2.1 Å), the local structure near C substitution is strongly distorted. As a result, the O atoms that connect with C are stretched away from the corresponding top-layer Ni (labeled as Ni #1) and one Ni–O bond breaks. Consequently, the coordination number of top-layer Ni decreases from 3 to 2, resulting in a new C-doped surface (denoted as C-surface, Fig. 4b). What is interesting for the C-surface is that the three C–O bonds have the same bond length of 1.30 Å (between the bond length of C–O and C=O), the angles between three O–C=O are the same and the centered C is on the same plane with the nearby three O. The information concluded that the C forms sp^2^ hybridization with three connected O, consistent with the observation of both C $1{\mathrm{s}} \to \pi _{{\mathrm{O}} - {\mathrm{C}} = {\mathrm{O}}}^ \ast$ and $1{\mathrm{s}} \to \sigma _{{\mathrm{O}} - {\mathrm{C}} = {\mathrm{O}}}^ \ast$ transitional signals in XANES spectra (Fig. 3b).

C-surface has two structural characteristics, Ni vacancy and C doping. Their effects on HER performance of NiO were studied separately. First, the investigation on Ni vacancy was made through the comparison between o-surface and p-surface, as o-surface has Ni vacancies while p-surface does not. After carefully considering all possible H adsorption sites on both surfaces, we noticed that the existence of Ni vacancy does not increase the number of active sites toward H adsorption per unit area of the o-surface compared with p-surface (detailed information is given in SI, Supplementary Figs. 7 and 8, and Supplementary Table 2). Then the effect of C doping was studied for both Ni vacancy resided C-surface and o-surface. It is known that the performance of catalysts is strongly related to their electronic structure, which can be tuned by dopants^26,27^. To gain an in-depth understanding of the electronic structure of C-surface, projected density of states (PDOS) of the C-surface structure were plotted, and compared with the PDOS of the o-surface (Fig. 4c, d). The PDOS plot shows that the C doping significantly reduces the band gap from ~1.5 to ~0.6 eV. This indicates that the conductivity of Ni_1−x_O was improved after C doping, which is beneficial for the electron transport in HER. Further analysis revealed that the narrowed band gap is caused by the downshifting of the majority (spin up) conduction band minimum (CBM) to below the Fermi level and overlapping with valence band maximum (VBM), moving the VBM closer to the Fermi level (Fig. 4c, d). The change of PDOS can be attributed to the C doping mediated change of the local structure of top-layer Ni. One of the three Ni (#1)–O bonds in o-surface was broken owing to the strong stretch applied by the short C–O bond nearby, which endows the top-layer Ni (#1′) on C-surface with higher electron density, thus, upshifts the VBM. The PDOS change of the top-layer Ni on o-surface (Ni #1) and C-surface (Ni #1′) also confirms the effect of C doping, because the majority (spin up) CBM shifts down to below the Fermi level as well, and mixes with the VBM, resulting in the VBM upshifting to around the Fermi level (Fig. 4e, f). The comparison of charge density mapping of the top-layer Ni sites (#1 and 1′) on o-surface and C-surface shows clear evidence that the electron density for the top-layer Ni (#1) was largely increased after C doping (Fig. 4g). Although carbon is a *n*-type doping, it did not change the NiO (Supplementary Fig. 9) from *p*-type to *n*-type. This is because the concentration of electrons is not enough to compensate the majority holes in the NiO lattice^28–30^, as evidenced by the nearly same work function before and after C doping (Supplementary Fig. 10). To quantify the charge density change of the top-layer Ni (#1 and 1′), the analysis of atomic charge difference (Δ*Q*) was performed according to the following equation, based on Bader charge partitioning scheme^31^,

1 $$\Delta Q = Q_{{\mathrm{surface}}} - Q_{{\mathrm{bulk}}}$$

where *Q*_surface_ is the amount of electrons carried by the surface ions, and *Q*_bulk_. is the amount of electrons of the corresponding ions in the bulk structure. Thus a larger Δ*Q* represents a higher electron density carried by the surface ion. The Δ*Q* for the top-layer Ni (#1′) from C-surface is 0.636, which is considerably larger than the value of 0.168 obtained from the Ni (#1) on o-surface, again confirming higher electron density on the top-layer Ni (#1′) on C-surface. The larger electron density on Ni sites is believed to be helpful for H adsorption, as Ni donates electrons to H in the Ni–H bond owing to the larger electronegativity of H (the electronegativity of Ni is 1.8, which is smaller than that of 2.1 of H).

### Hydrogen adsorption profile on C–Ni_1−x_O

**Fig. 5:**
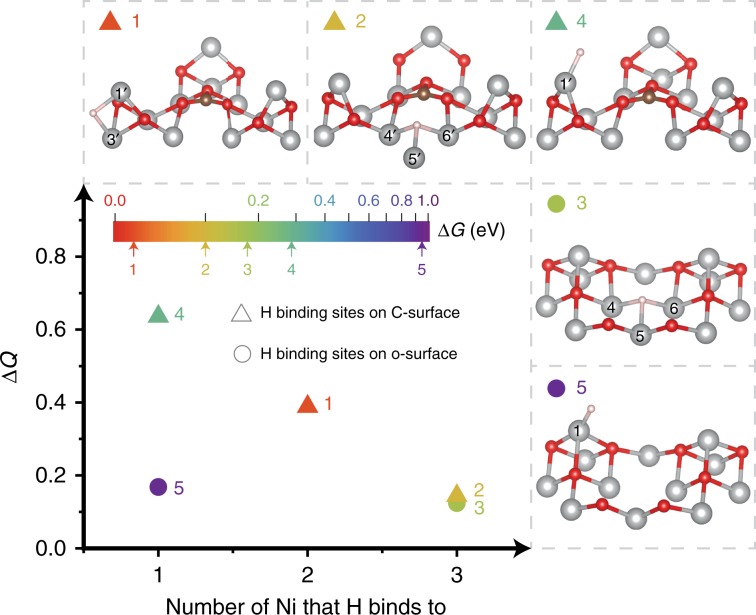
Correlation between atomic charge difference (Δ*Q*), number of Ni that H binds to and Gibbs free energy change of H adsorption (Δ*G*_H_). The 5 points represent five different H adsorption sites on o-surface and C-surface.

Compared with o-surface, carbon doping not only enhances the H adsorption activity of previously existed sites, but also exposes newly active H adsorption sites (Supplementary Figs. 8 and 11). Specifically, the improvement of H adsorption activity over old sites can be concluded by comparing the H adsorption onto single-fold sites (Ni #1 and #1′) or threefold hollow sites. For example, Δ*G*_H_ of the top-layer Ni (#1′) on C-surface (structure 4 in Fig. 5) has a much smaller value of 0.282 eV than the value of 0.935 eV obtained from the identical Ni (#1) on o-surface (structure 5 in Fig. 5), as suggested by the PDOS, the charge density mapping and Δ*Q* analysis. In addition, the hollow sites on C-surface (Ni # 4′, 5′, and 6′, structure 2 in Fig. 5) also show a smaller Δ*G*_H_ value of 0.104 eV compared with the value of 0.152 eV obtained from the identical hollow sites on o-surface (Ni # 4, 5, and 6, structure 3 in Fig. 5). On the other hand, the newly exposed bridge sites (Ni #1′ and 3′, structure 1 in Fig. 5) of the C-surface exhibit an almost thermoneutral Δ*G*_H_ value of 0.031 eV, indicating that the introduced new sites by carbon doping is favorable for the adsorption of H.

Furthermore, our analysis also showed that Δ*G*_H_ strongly depends on the Δ*Q* of Ni as well as the number of Ni that H bonds to. A plot of the change of Δ*G*_H_ against these two variables is depicted in Fig. 5. The comparison between one-fold Ni sites (e.g., Ni #1′ and Ni #1 in structure 4 and 5, respectively) shows that structure 4 with larger Δ*Q* exhibits smaller Δ*G*_H_ (0.282 eV) than structure 5 (0.935 eV). This is because the higher electron density on Ni site makes it easier for H to bind with. When the structures (e.g., structures 3 and 5) have similar Δ*Q*, increasing the number of Ni that H bonds to can largely shift down the Δ*G*_H_ from 0.935 to 0.152 eV, which is due to the stronger interaction between H and multi-fold Ni. Similar trend was observed by comparing structure 2 with structure 5. This finding suggests that increased electron density or multi-folds of H binding sites are the underlying reasons for the easier adsorption of H on Ni sites in Ni_1−x_O system.

### HER performances

**Fig. 6:**
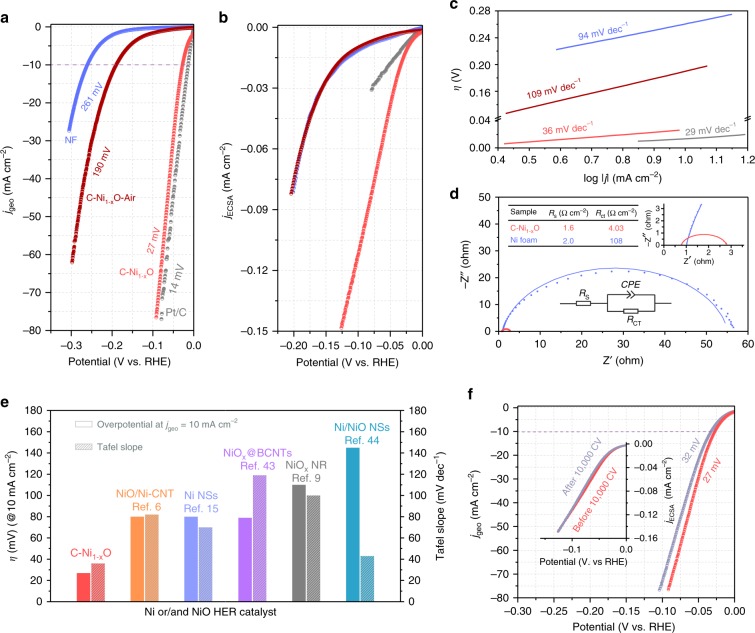
HER performances of C–Ni_1−x_O. **a** HER polarization curves obtained from NF, C–Ni_1−x_O–Air, C–Ni_1−x_O, and Pt/C in 1.0 M KOH saturated with nitrogen at a scan rate of 1 mV s^−1^. **b** Polarization curves of Pt/C, NF, C–Ni_1−x_O–Air, and C–Ni_1−x_O with current normalized to ECSA. **c** Tafel plots of the NF, C–Ni_1−x_O–Air, C–Ni_1−x_O, and Pt/C. **d** Electrochemical impedance spectra of the NF and C–Ni_1−x_O measured at the potential of −0.15 V vs RHE, with frequency ranging from 100 kHz to 1 Hz and an amplitude of 5 mV. Dots and solid lines are the experimental data and simulated results based on the equivalent circuit illustrated in the inset, respectively. **e** The comparison of the overpotential at 10 mA cm^−2^ and Tafel slopes of C–Ni_1−x_O with the reported state-of-the-art Ni- and NiO-based HER catalysts, including NiO/Ni-CNT^6^, Ni nanosheets (NSs)^15^, NiO_x_@Bamboo-like CNTs (BCNTs)^43^, NiO_x_ nanorods (NRs)^9^, and Ni/NiO NSs^44^. **f** Polarization curves of the C–Ni_1−x_O collected before and after 10,000 cycles. Inset figure shows the polarization curves with current normalized to ECSA. All of the data here are *iR* corrected.

The HER performances of C–Ni_1−x_O were characterized in 1.0 M KOH saturated with nitrogen through linear sweep voltammetry (LSV) at a scan rate of 1 mV s^−1^ (Fig. 6a). A control sample without carbon doping was prepared by annealing C–Ni_1−x_O in air (denoted as C–Ni_1−x_O–Air, Supplementary Fig. 12 and 13, and Supplementary Note 3). C–Ni_1−x_O–Air, NF, and Pt/C (10 wt% Pt) were also measured under the same condition for comparison. Prior to the measurement, all of the electrodes were conditioned through cyclic voltammetry to wet the electrode structure (see Methods). XPS and XANES characterizations (Supplementary Fig. 14 and Supplementary Note 4) confirmed that the conditioning did not change the chemical nature of C–Ni_1−x_O. It is noteworthy that NiO is the thermodynamically stable phase in the potential window between 0 and −174 mV vs RHE according to the NiO pourbaix diagram^32^. The presence of overpotential of NiO reduction can further expand this potential window^8^. Significantly, C–Ni_1−x_O achieved an ultralow overpotential of 27 mV at the geometric current density (*j*_geo_) of 10 mA cm^−2^, which is comparable with the 14 mV of the benchmark Pt/C catalyst at the same current density. Supplementary Fig. 15 shows the polarization curves and statistical plot of the overpotentials obtained from four different C–Ni_1−x_O samples, and they have comparable performance with an average overpotential of 29 ± 1.8 mV at *j*_geo_ = 10 mA cm^−2^. The performance comparison between NF and C–Ni_1−x_O excludes the substrate contribution to the ultralow overpotential. C–Ni_1−x_O–Air exhibited an overpotential of 190 mV at *j*_geo_ = 10 mA cm^−2^. Although this value is better than that of Ni foam (260 mV), it is considerably worse than the performance of C–Ni_1−x_O. The result suggests that the improved overpotential of C–Ni_1−x_O is because of carbon doping, which is consistent with the simulation results. The total electrode activity is determined by two major factors, the intrinsic activity of the catalyst and the quantity of active sites (or the electrochemical surface area, ECSA) that is electrolyte accessible^33^. To evaluate the intrinsic activity of C–Ni_1−x_O, its current was normalized to ECSA (Fig. 6b and Supplementary Fig. 16). Significantly, C–Ni_1−x_O still showed substantially larger HER current density than that of C–Ni_1−x_O–Air, NF, and Pt/C at the same overpotentials under the measurement conditions. According to our DFT calculations, the excellent intrinsic activity of C–Ni_1−x_O can be attributed to the improved H adsorption activity of Ni sites as a result of carbon doping. In addition, Tafel plot provides important information on the rate limiting step of HER. As shown in Fig. 6c, C–Ni_1−x_O exhibits a Tafel slope of 36 mV dec^−1^, which is comparable with the 29 mV dec^−1^ of Pt/C but much smaller than that of C–Ni_1−x_O–Air (109 mV dec^−1^) and NF (94 mV dec^−1^). This small Tafel slope value suggested that the Heyrovsky step ($\ast {\mathrm{H}} + {\mathrm{H}}_2{\mathrm{O}} + {\mathrm{e}}^ - \rightleftharpoons \ast {\mathrm{H}}_2 + {\mathrm{OH}}^ -$), in which water molecules are dissociated to provide protons for the generation of dihydrogen, is the rate limiting step. The enhanced HER performances of C–Ni_1−x_O was also evidenced by the small charge transfer resistance (*R*_ct_ = 4.03 Ω cm^−2^), which is almost 27 times lower than that of NF (*R*_ct_ = 108 Ω cm^−2^), indicating the efficient electron transfer kinetics C–Ni_1−x_O during HER process (Fig. 6d, and Supplementary Table 3). In comparison to other Ni- and NiO-based HER catalysts, C–Ni_1−x_O exhibits considerably smaller overpotential (at *j*_geo_ = 10 mA cm^−2^) and Tafel slope (Fig. 6e and Supplementary Table 4). Furthermore, the C–Ni_1−x_O catalyst showed excellent stability at both low (5 mA cm^−2^) and high (60 mA cm^−2^) current densities (Supplementary Fig. 17). The initial current drop in the first two hours (Supplementary Fig. 17) is possibly due to the dynamic process of reaching an equilibrium between gas evolution and electrolyte diffusion. The current level became stable after achieving the dynamic equilibrium. Notably, the same current drop profile and the same current level were observed after the replacement of electrolyte (60–135 h, Supplementary Fig. 17a). This is a direct evidence that this current drop is not stemmed from catalyst’s compositional change or active site failure. An accelerated degradation measurement was also performed through cyclic voltammetry (CV) for 10,000 cycles at a scan rate of 100 mV s^−1^ (Fig. 6f). The total electrode activity is comparable before and after 10,000 cycles, with an overpotential of 27 mV slightly increased to 32 mV at the *j*_geo_ = 10 mA cm^−2^ after the test. It is noteworthy that the intrinsic activity (*j*_ECSA_) remains the same before and after the 10,000 cycles (inset of Fig. 6f).

### Water dissociation pathway on C–Ni_1−x_O

**Fig. 7:**
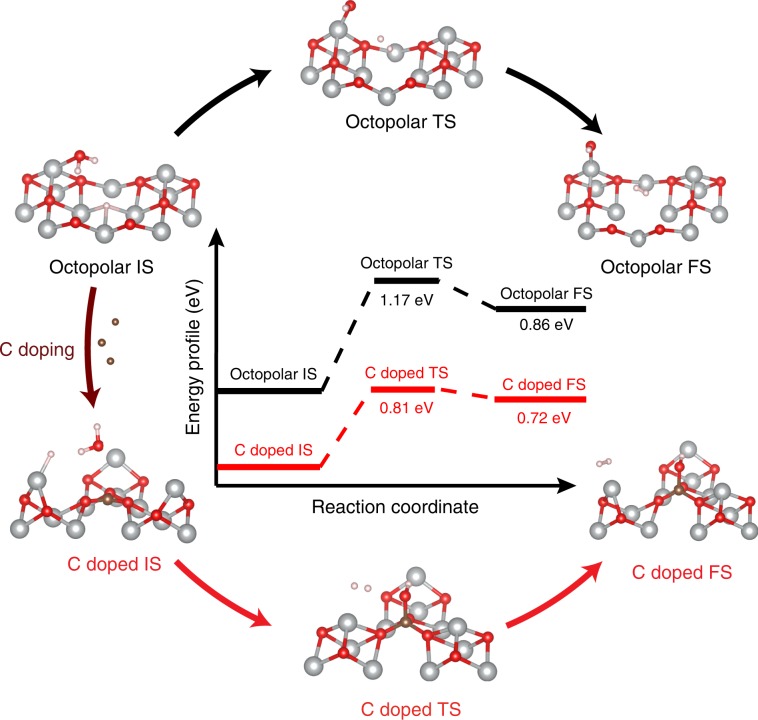
HER energy profile. The reaction energy profile of the Heyrovsky step for o-surface and C-surface. The corresponding IS, TS, and FS structures for o-surface and C-surface are listed in the surrounding circle.

The Tafel plot indicates that the Heyrovsky step is the rate limiting step for C–Ni_1−x_O HER catalyst. Therefore, we performed water dissociation energy barrier calculation based on the Heyrovsky step for both o-surface and C-surface. All of the initial state (IS), transition state (TS) and final state (FS) structures for both surfaces (Supplementary Figs. 18 and 19, and Supplementary Table 5) in Heyrovsky step were shown together with their energy profile (Fig. 7). For o-surface, initially, H_2_O approached to the top-layer Ni due to the Van der Waals interaction while a H atom bonds to the adsorption favorable hollow sites of the third-layer Ni (Octopolar IS). Subsequently, H–OH bond was cleaved simultaneously with the formation of Ni–OH bond, which is beneficial for lowering the energy of the H_2_O/o-surface system (Octopolar TS, structural details are given in Supplementary Fig. 20). The accompanied energy barrier of the Heyrovsky step on o-surface was calculated to be 1.17 eV. On the other hand, C-surface has a completely different reaction pathway. For instance, H_2_O was found to preferably stay on the top of carbon dopant through the strong affinity of carbon towards oxo groups (C-doped IS). This observation is consistent with our hypothesis that carbon is a water adsorption site. H_2_O was then dissociated with the assistance of the carbon dopant in the TS (structural details can be seen in Supplementary Fig. 21), which exhibits a lower activation energy barrier of 0.81 eV compared with the 1.17 eV of the o-surface. The lowered energy barrier of Heyrovsky step on C-surface could be attributed to the unique C–O_3_ local structure. Since the carbon dopant forms sp^2^ hybridization structure with the nearby three oxygen, delocalized electrons of the *π* bond in this C–O_3_ structure increases the electron density around the carbon center. Combined with the vertical orientation characteristic of the p_z_ orbital of carbon dopant, it can be expected that the overlapping of the p_z_ orbital of carbon with the hybridized p orbital of O (in H_2_O) could be facilitated, which helps form a strong C–OH bond and release more energy. This is also supported by our calculated results that the C–OH bond energy of 504 kJ mol^−1^ in TS of C-surface is higher than the 448 kJ mol^−1^ bond energy of Ni–OH in the TS of o-surface. These calculations provide important insights into the favorable HER reaction pathway on Ni_1−x_O and clarify that carbon dopant, due to the unique C–O_3_ local sp^2^ hybridization structure, is the hot-spot for water dissociation.

## Discussion

In summary, we have demonstrated an effective NiO-based catalyst for water-alkali HER. DFT simulations reveal that carbon dopant distorts the local structure of NiO and decreases the coordination number of the top-layer Ni (#1). PDOS plot, charge density mapping and atomic charge difference analysis all indicate higher electron density around the top-layer Ni (#1′) on carbon doped surface, with Δ*G*_H_ calculation further confirming the top-layer Ni (#1′) and the nearby bridge site serving as newly exposed hot spots for H adsorption. DFT calculations also showed that carbon dopant, due to the unique C–O_3_ local sp^2^ hybridization structure, serves as an active site to facilitate the dissociation of H_2_O molecule with a lower energy barrier of 0.81 eV compared with 1.17 eV of the surface without carbon doping. As a result, this carbon doped Ni_1−x_O exhibits an ultralow overpotential of 27 mV at the geometric current density of 10 mA cm^−2^ and a low Tafel slope of 36 mV dec^−1^, outperforming previously reported nickel oxide and other nickel-based alkaline HER catalysts. Most importantly, this work exemplified how to activate an important water-alkali HER electrocatalyst through rational doping. Other *n*-type dopants such as Si, N, and P with smaller atomic size than Ni could have a similar effect as C dopant in tuning the electron density and the H adsorption energy of Ni sites. We believe these findings also provide important design guidance for other water-alkali HER electrocatalysts.

## Methods

### Preparation of carbon doped Ni_1−x_O on NF

A piece of NF (bulk density of 350 g m^−2^, Hefei Kejing Materials Technology Co. Ltd, China) was anodized in a two-electrode system using a piece of Ni foil as the counter electrode in 0.3 M oxalic acid (Thermo Fisher Scientific, product no. 171514) solution. The anodization was carried out at the temperature of −5 °C at a constant voltage of 50 V for 10 min. The anodized NF (nickel oxalate/NF) was then rinsed thoroughly with DI water and ethanol, successively. The anodized samples were then dried in vacuum at 100 °C for 1 h, and subsequently annealed in Ar atmosphere (ultrahigh purity 99.998%) at 400 °C for 40 min. The control sample was prepared by annealing C–Ni_1−x_O in air at 400 °C for 10 min.

### Structural characterizations

The morphology of NiC_2_O_4_·2H_2_O bulk crystals and carbon doped Ni_1−x_O nanorods grown on NF were determined by scanning electron microscopy (SEM, Hitachi S-4800 II). Crystal structure and elemental mapping were characterized on the carbon doped Ni_1−x_O particle subunit through TEM (Talos F200X). The local coordination environment of C and O in carbon doped Ni_1−x_O/NF was characterized by X-ray absorption spectroscopy (XAS) at the C K-edge and O K-edge, respectively. X-ray absorption spectroscopy spectra were measured on Beamline 8.0.1 at the Advanced Light Source (ALS), Lawrence Berkeley National Laboratory (LBNL). Energy resolution was set to 0.2 eV for C and O K-edge XAS spectra, respectively. All spectra were normalized to the incident photon flux with careful energy calibrations to the known reference samples. All spectra were recorded in the total electron yield (TEY) and total fluorescence yield (TEY) detection modes simultaneously in the XAS experimental chamber, which has the base pressure of better than 1.0 × 10^−9^ torr. In order to avoid the intensive signal interference from the NF, the powders collected from the anodization were used for XRD (Rigaku SmartLab) and XPS (Thermo Scientific ESCALAB 250Xi) analysis. Thermogravimetric analysis (TA 500 Thermoanalyze) was performed in Ar atmosphere from room temperature to 450 °C with a ramping rate of 10 °C min^−1^.

### Pt/C electrode preparation

Five milligrams of the Pt/C (10 wt% of Pt) was dispersed in the mixture of 958 μL ethanol and 20 μL of DI H_2_O. Twenty-two microliters of Nafion (5 wt%) was added as the binder. Subsequently, the mixture was sonicated for 30 min to well disperse the catalyst powders. Pt/C ink was drop cast on the glassy carbon electrode with an areal mass loading of 1 mg cm^−2^ and dried in air.

### Electrochemical measurement

The electrochemical performances were investigated in a three-electrode system, with Hg/HgO (1 M KOH, Thermo Fisher Scientific, product no. 178481) and graphite rod as the reference electrode, and counter electrode, respectively. Before measurement, Hg/HgO reference electrode was corrected against reversible hydrogen electrode (RHE) based on the literature reported method^34^. The HER performances data were collected in nitrogen saturated 1.0 M KOH (Thermo Fisher Scientific, product no. 178481) electrolyte. All of the working electrode were CV conditioned from 0.33 to −0.32 V vs RHE at a scan rate of 50 mV s^−1^ for 50 cycles to ensure the enough wetting of electrode first, followed by an LSV measurement at a scan rate of 1 mV s^−1^. Electrochemical impedance spectroscopy (EIS) was performed at the potential of −0.15 V vs RHE, with frequency from 100 kHz to 1 Hz and an amplitude of 5 mV. The LSV was *iR* corrected based on the EIS results. Mott-Schottky measurements were performed on the C–Ni_1−x_O at a frequency of 1000 Hz under a stable open circuit potential of −0.03 V vs Hg/HgO.

### DFT simulation

Density functional theory calculations were performed with plane-wave basis codes Quantum Espresso (QE), with exception for transition state calculations which were carried out using the plane-wave basis code Vienna Ab Initio Simulation Package (VASP). In all calculations, Perdew–Burke–Erzenhof exchange and correlation functional with Hubbard U correction (PBE + U) was employed^35^. An effective Hubbard U value of 5.3 eV was used as reported in previous literature^36^. Ultrasoft pseudopotential from GBRV was used with a wavefunction cutoff of 40 Ry and charge density cutoff of 240 Ry^37^. In order to obtain the accurate energy barriers, Nudged Elastic Band (NEB) calculation was first performed to get the approximate saddle point, followed by the further convergence by DIMER calculation^38,39^. The vibrational frequencies for zero point energy and entropy were computed by Density Functional Perturbation Theory (DFPT)^40^ in Quantum Espresso, and an implicit solvation model^41,42^ was adopted to include the effect of solvent around solid surfaces. More computational details can be found in Supplementary Methods.

## Supplementary information

Supplementary information

## Data Availability

The data that support the plots of the article are available within this paper and corresponding supporting information. Other findings of this study are available from the corresponding authors upon reasonable request.

## Code availability

Computer codes that used to generate results that are reported in the paper are available from the corresponding authors upon reasonable request.
