# Supplementary material for: Carbon doping switching on the hydrogen adsorption activity of NiO for hydrogen evolution reaction
Source: Nat Commun. 2020 Jan 30;11:590. doi: 10.1038/s41467-020-14462-2 (PMC6992690; doi:10.1038/s41467-020-14462-2)
Supplement: Supplementary file 1 — Supplementary information [file 41467_2020_14462_MOESM1_ESM.pdf]

Supplementary Information

Carbon Doping Switching on the Hydrogen Adsorption Activity of NiO for Hydrogen Evolution Reaction

Kou *et al*.

Supplementary Figures

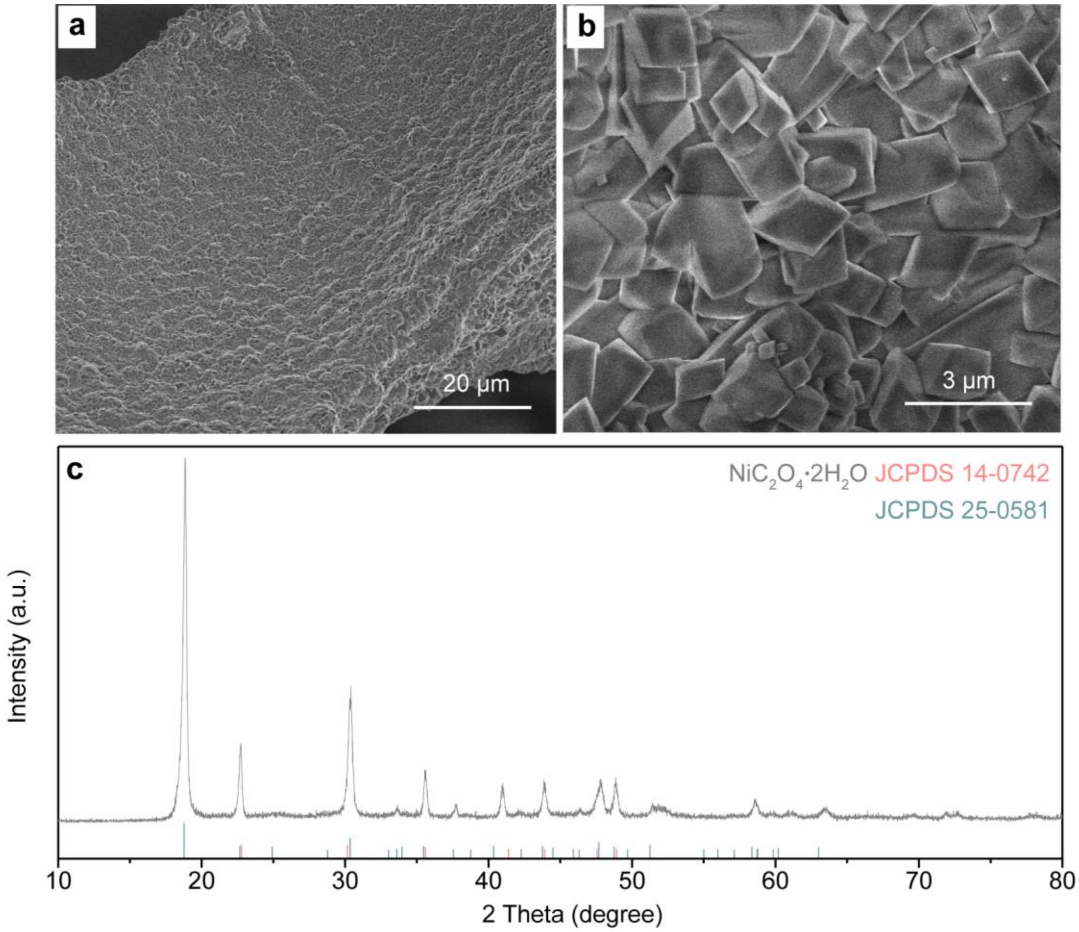

Supplementary Figure 1. Structural characterization of $NiC_2O_4·2H_2O$ a-b, SEM images of $NiC_2O_4·2H_2O$ grown on NF. **c**, XRD pattern of $NiC_2O_4·2H_2O$ powder.

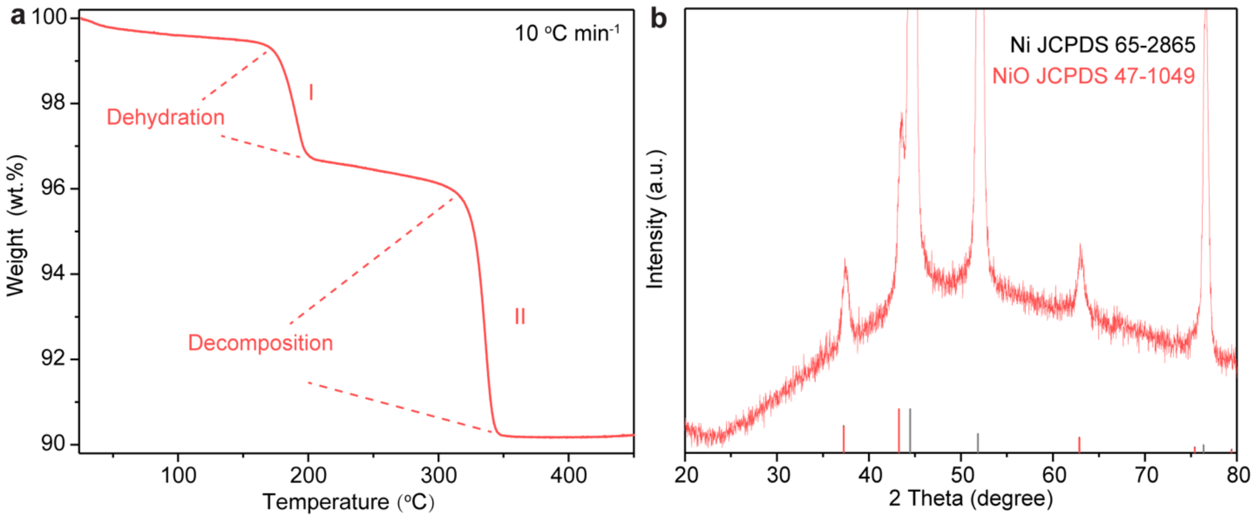

Supplementary Figure 2. TGA of $NiC_2O_4{\cdot}2H_2O$ and phase composition of C-$Ni_{1-x}O$ a, TGA of the $NiC_2O_4{\cdot}2H_2O$ in argon atmosphere with a ramping rate of 10 ^{o}C min^{-1}. **b**, XRD pattern obtained from the C-$Ni_{1-x}O$ powder.

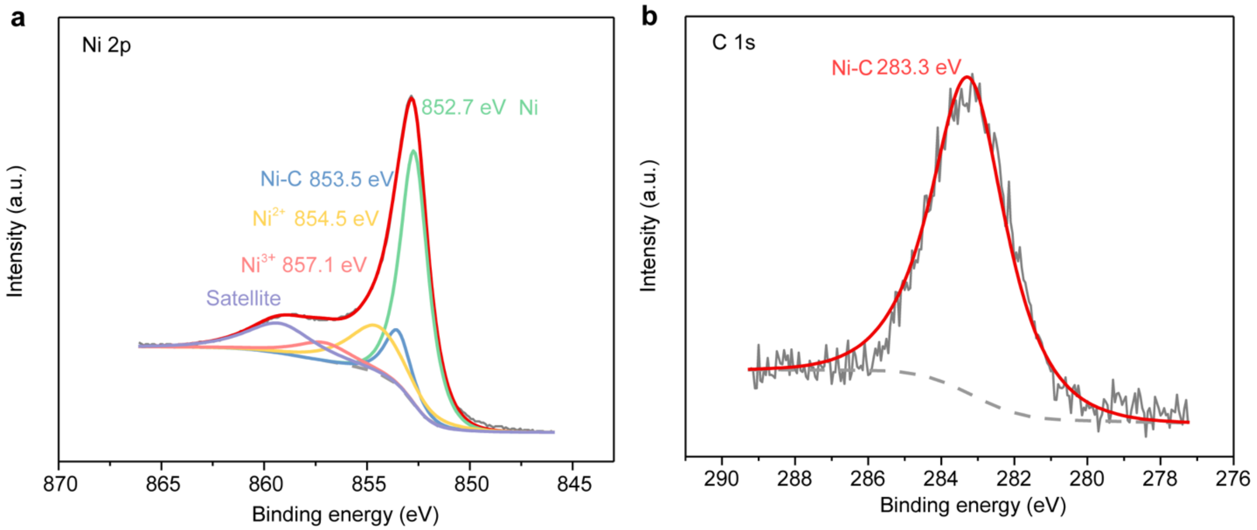

Supplementary Figure 3. XPS of annealed $NiC_2O_4·2H_2O$ after argon plasma etching a-b, Ni 2p and C 1s XPS.

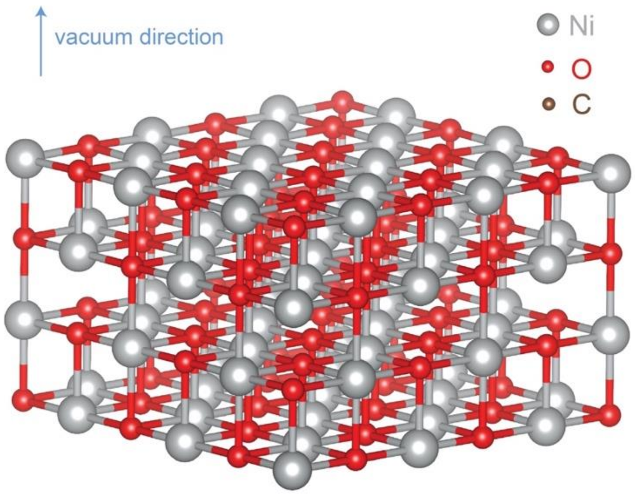

Supplementary Figure 4. The structure of (100) surface of NiO The vacuum's direction is given in the figure. The bottom layer is fixed (the color coding for different atoms is consistent for the whole paper).

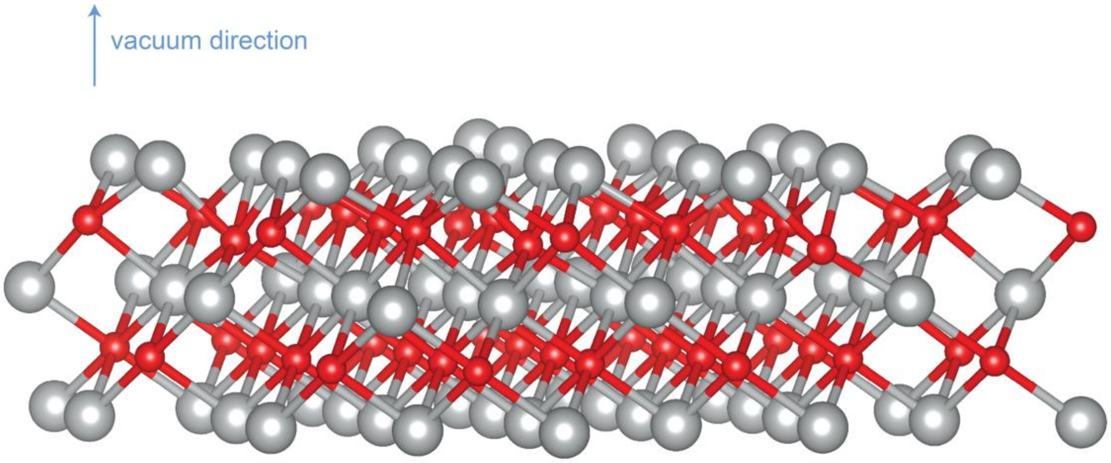

Supplementary Figure 5. Ni terminated (111) surface of NiO The vacuum's direction is given in the figure.

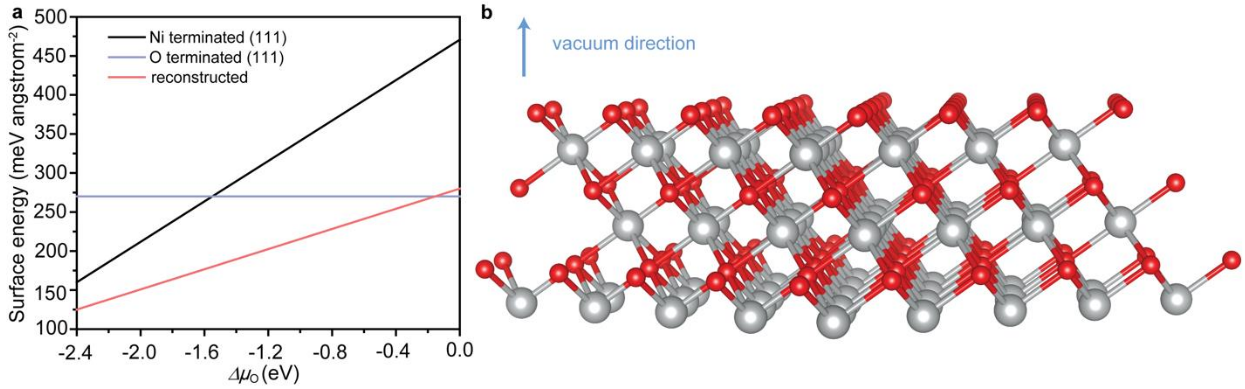

Supplementary Figure 6. NiO (111) surface phase diagram and structure a, The surface phase diagram for (111) surface of NiO. **b**, The structure of O terminated (111) surface. The structures of reconstructed surface and Ni terminated (111) are given in Figure 4a and Supplementary Figure 5, respectively.

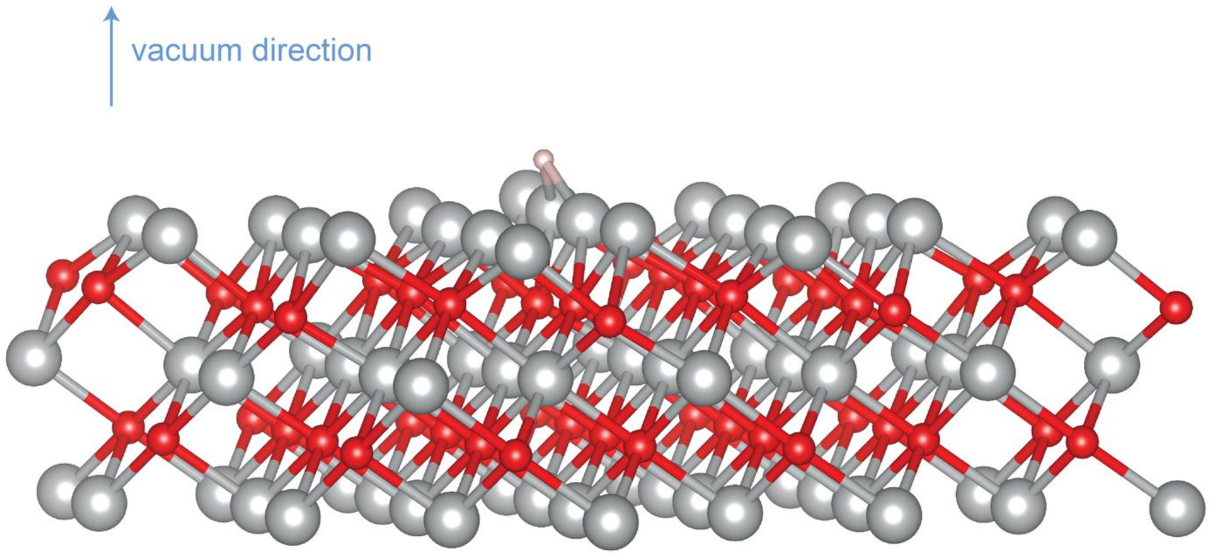

Supplementary Figure 7. The structure for the two-folds H adsorption site on p-surface The direction of vacuum is given in the figure.

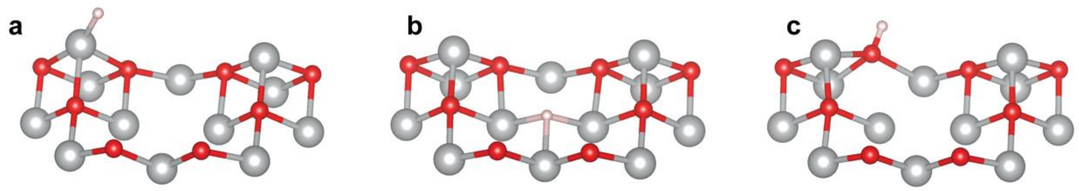

Supplementary Figure 8. The structure for the H adsorption sites on o-surface a, Ni #1, **b**, the hollow site for the third layer Ni sites, **c**, the second layer O sites. Only the first three layers' atoms are shown here.

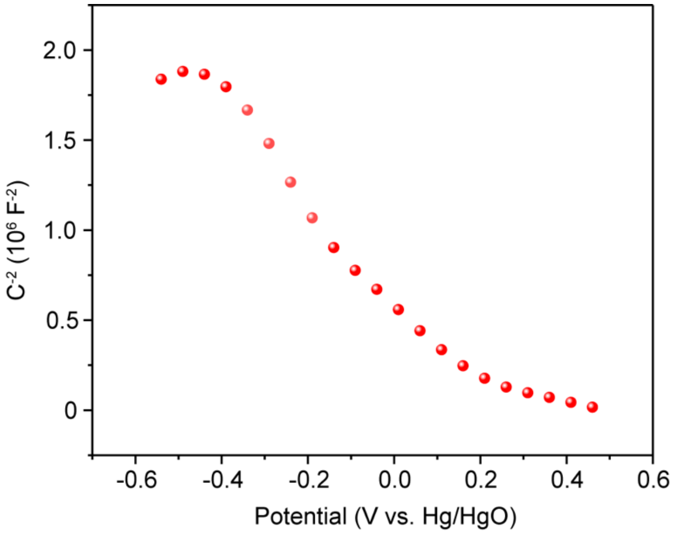

Supplementary Figure 9. Mott-Schottky plot of the C doped NiO The curve was collected in 1.0 M KOH at a frequency of 1000 Hz under an open circuit potential of -0.03 V vs. Hg/HgO.

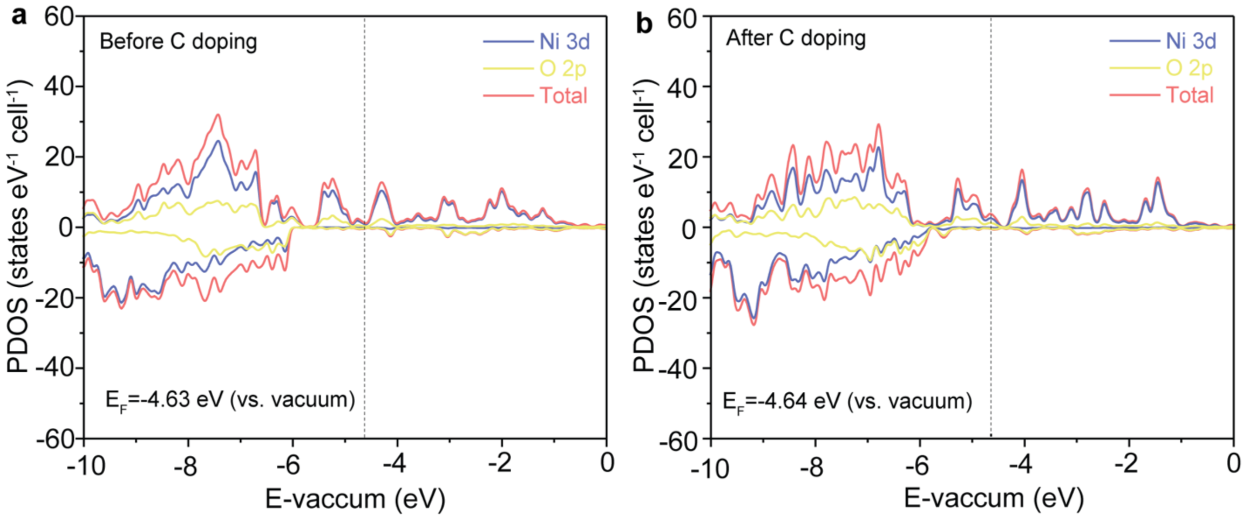

Supplementary Figure 10. PDOS plots of NiO a-b, PDOS plots of NiO before and after C doping. The eigenvalues are absolute values referenced to the vacuum level.

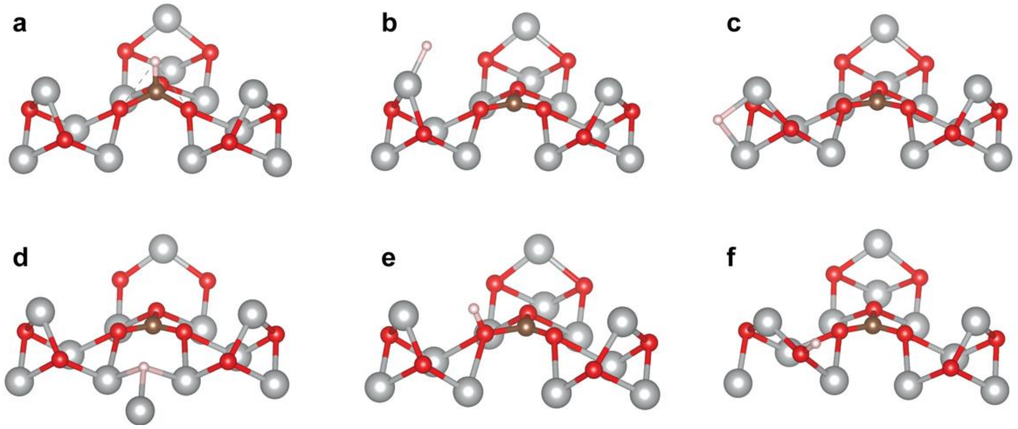

Supplementary Figure 11. The structure for the H adsorption sites on C-surface H bonds with **a**, the dopant C, **b**, Ni #1', **c**, Ni #1' and one third layer Ni, **d**, hollow site of the third layer Ni, **e**, O which connects with dopant C, and **f**, O which connects with Ni #1'. Only the first three layers' atoms are shown here.

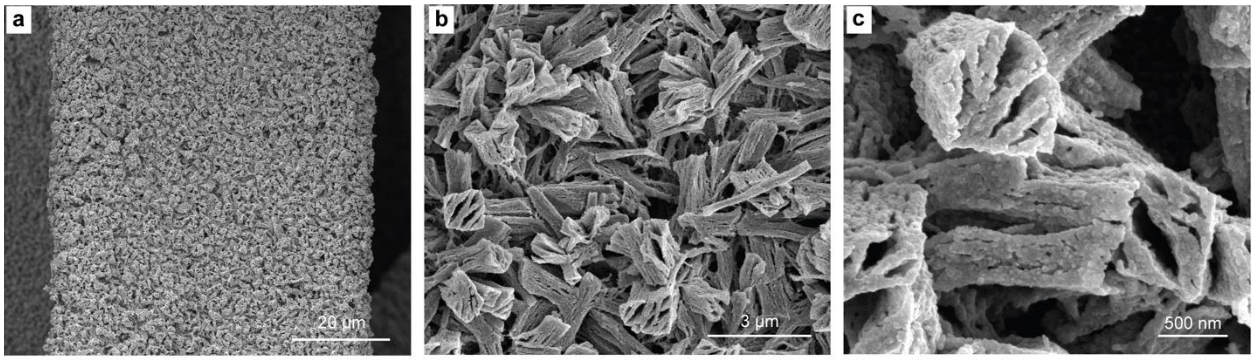

Supplementary Figure 12. Morphology of C-Ni$_{1-x}$O-Air/NF a-c, SEM images of the C-Ni$_{1-x}$O-Air/NF collected at different magnifications.

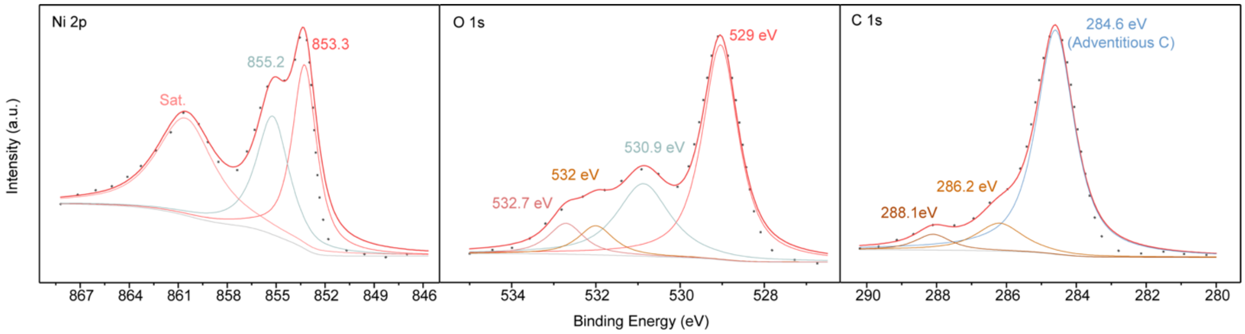

Supplementary Figure 13. XPS spectra of C-Ni_{1-x}O-Air Ni 2p, O 1s, and C 1s XPS spectra of C-Ni_{1-x}O-Air powder sample.

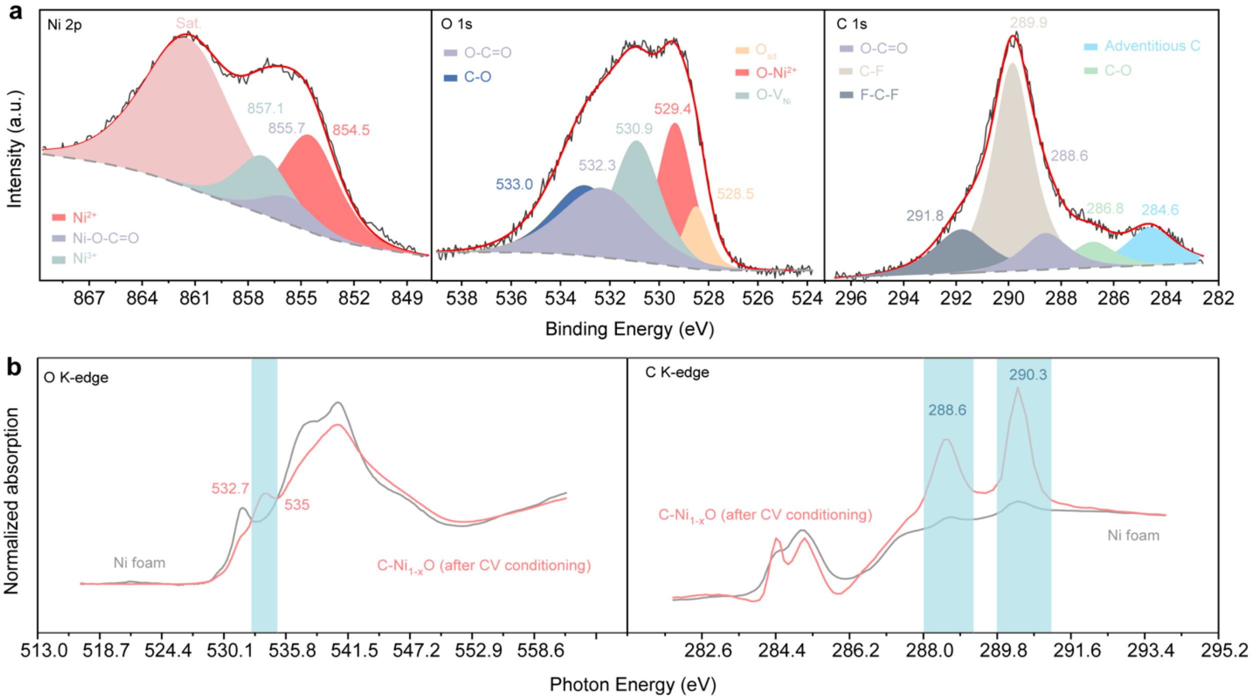

Supplementary Figure 14. Chemiacl environment of C-$Ni_{1-x}O$ after CV conditioning a, Ni 2p, O 1s, and C 1s XPS spectra of the C-$Ni_{1-x}O$ powder sample after CV conditioning. **b**, O K-edge and C K-edge XANES spectra of the C-$Ni_{1-x}O$/NF (red curve) after CV conditioning and NF (grey curve).

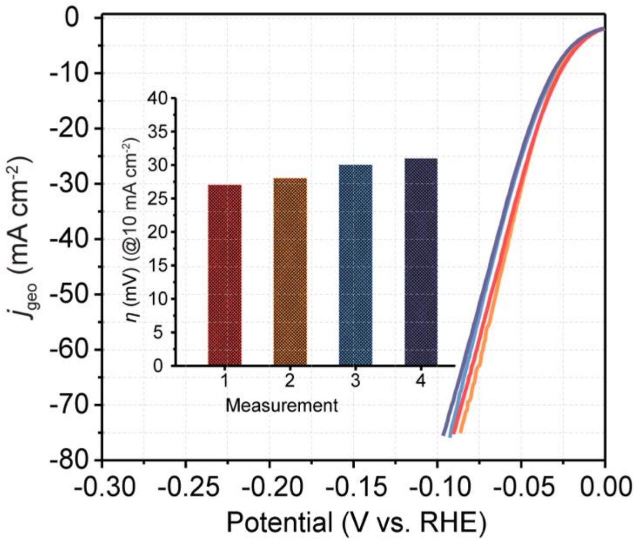

Supplementary Figure 15. Polarization curves of four independent C-$Ni_{1-x}O$ samples The samples were measured in the same condition at the scan rate of 1 mV s^{-1} in nitrogen saturated 1.0 M KOH (*iR* corrected). Inset is the overpotential at the j_{geo}=10 mA cm^{-2} of the four samples.

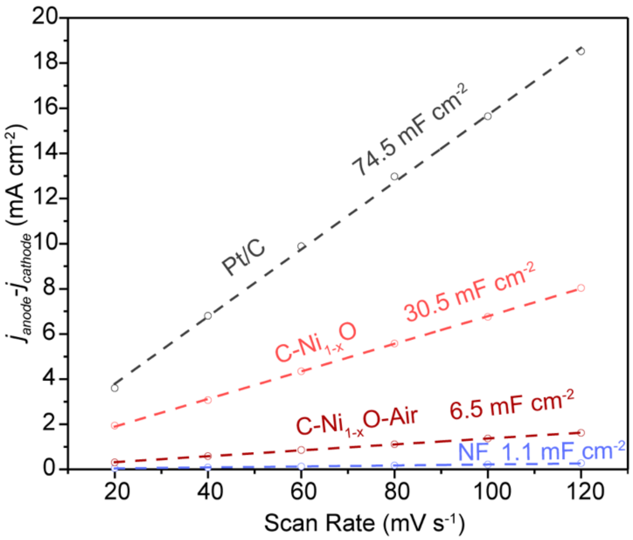

Supplementary Figure 16. Areal capacitance of different samples The difference of the anodic and cathodic current density difference of C-Ni_{1-x}O, C-Ni_{1-x}O-Air, NF, and Pt/C versus scan rate.

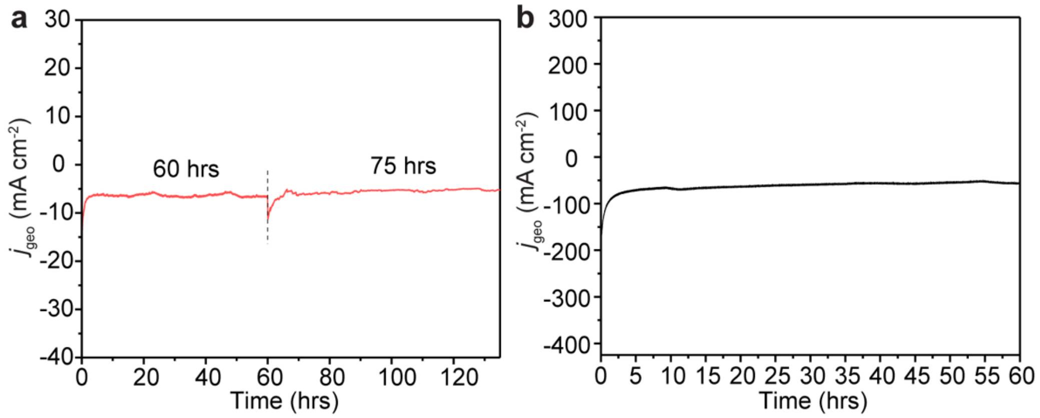

Supplementary Figure 17. Long term stability of C-$Ni_{1-x}O$ a-b, Chronoamperometry curves of the C-$Ni_{1-x}O$ samples at the potential of -32 mV and -275 mV vs. RHE (without *iR* correction), respectively. Dashed line represents the replacement of the electrolyte.

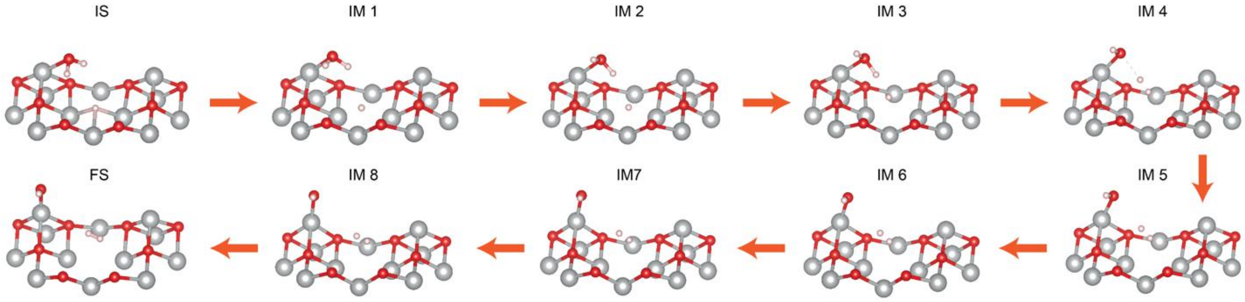

Supplementary Figure 18. The IMs of the Nudged Elastic Band (NEB) calculation on o-surface The calculation is for Heyrovsky step. IMs are the images, IS is the initial state, FS is the final state and DIMER calculation was further performed on IM4 to find the saddle point. Only the first three layers' atoms are shown here.

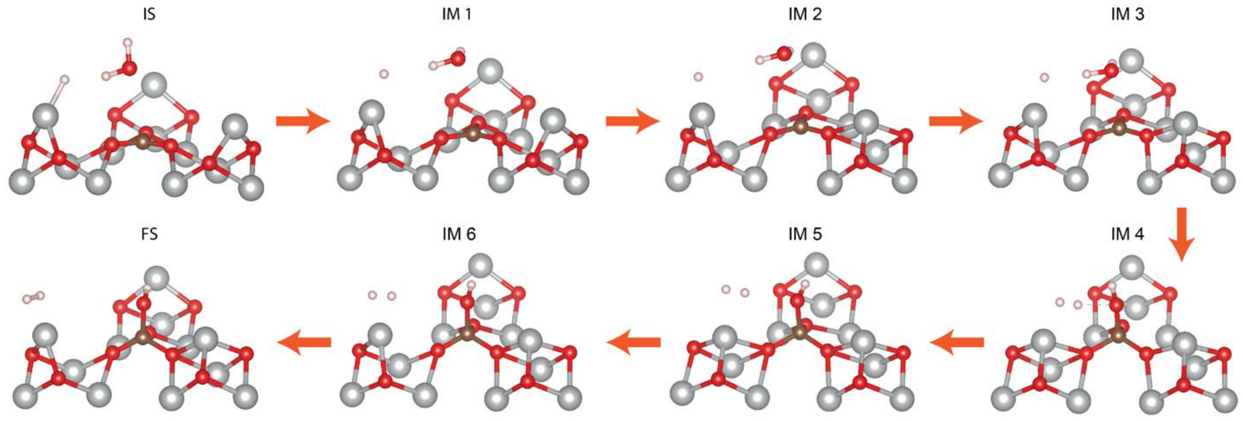

Supplementary Figure 19. The IMs of the Nudged Elastic Band (NEB) calculation on C-surface The calculation is for Heyrovsky step. IMs are the images, IS is the initial state, FS is the final state and DIMER calculation was further performed on IM4 to find the saddle point. Only the first three layers' atoms are shown here.

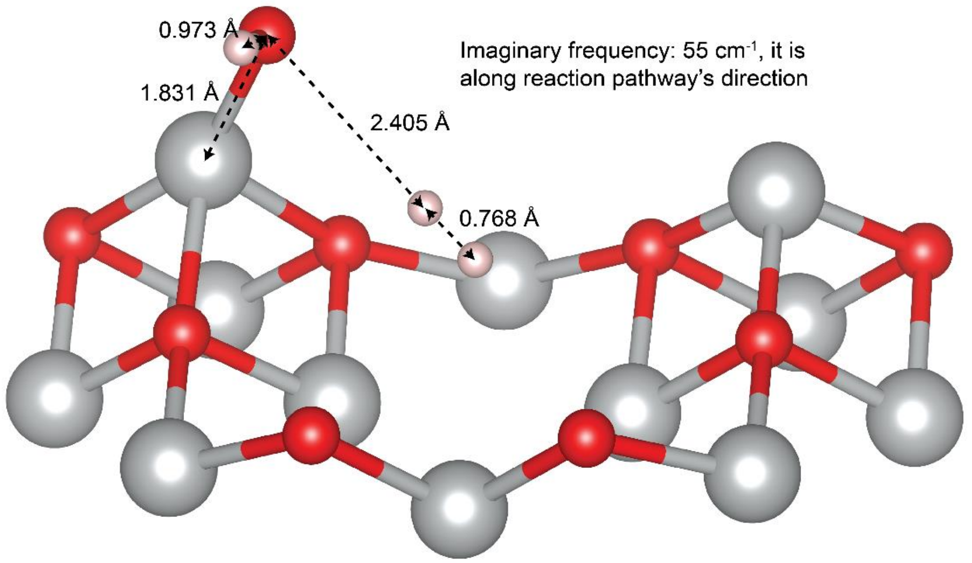

Supplementary Figure 20. The magnified transition state (TS) of o-surface Only the first three layers' atoms are shown here.

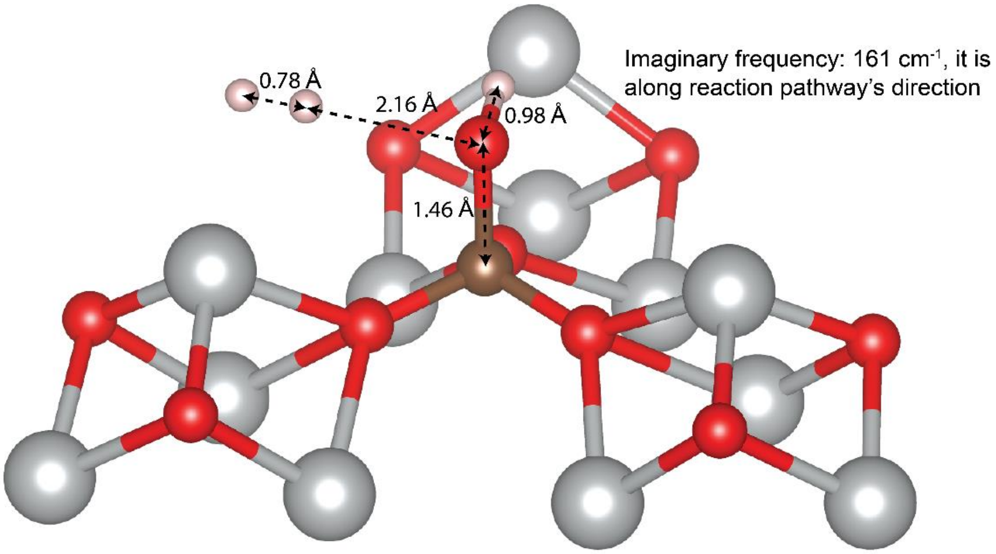

Supplementary Figure 21. The magnified TS of C-surface Only the first three layers' atoms are shown here.

Supplementary Tables

Supplementary Table 1. Properties comparison Lattice constant, atomic magnetic moment, and band gap comparisons between literature experimental and calculation results, and our calculated results.

Properties	Experimental value	Literature (GGA + U, U_{eff} = 5.3 eV)	Our results (PBE + U, U_{eff} = 5.3 eV)
Lattice constant (Å)	4.17 (Ref. [1])	4.19 (Ref. [2])	4.22
Atomic magnetic moment (μ_B)	1.64 (Ref. [3])	1.69 (Ref. [2])	1.64
Band gap (eV)	4.2 (Ref. [1])	3.1 (Ref. [2]); 2.43 (Ref. [4])	2.74

Supplementary Table 2. The summary of the total energy difference (ΔE), solvation energy difference between H bound surface and bare surface ($\Delta E_{solvation}$), vibration energy ($E_{vibraion}$), ΔG, atomic charge difference (ΔQ) of bound atom and coordination number (CN) of bound atom for all H binding sites

Surface	H binding site	$\Delta E(H)$ (eV)	$\Delta E_{solvation}(eV)$	$E_{vibraion}$ (eV)	$\Delta G(H)$ (eV)	ΔQ of bound atom	CN of bound atom
p-surface	Ni-H	-0.462	0.112	0.158	-0.192	0.650	3
o-surface	Ni-H (a)	0.768	-0.025	0.192	0.935	0.168	3
o-surface	Ni-H (b)	-0.034	0.013	0.173	0.152	0.124	5
o-surface	O-H (c)	-0.405	-0.014	0.321	-0.098	-0.129	4
C-surface	C-H (a)	1.808	0.007	0.322	2.137	N/A	3
C-surface	Ni-H (b)	0.108	0.007	0.167	0.282	0.636	2
C-surface	Ni-H (c)	-0.016	-0.087	0.134	0.031	0.389	2 and 5
C-surface	Ni-H (d)	0.013	-0.039	0.130	0.104	0.084	5
C-surface	O-H (e)	0.539	-0.100	0.307	0.746	0.515	3
C-surface	O-H (f)	-0.965	0.044	0.319	-0.602	-0.144	4

Supplementary Table 3. The simulated equivalent circuit results of the NF and C-$Ni_{1-x}O$ samples

Sample	R_s [Ω]	R_{ct} [Ω]	CPE-T	CPE-P
NF	1.0	54	1.751×10^{-4}	0.9125
C-$Ni_{1-x}O$	0.78	2.0	4.496×10^{-3}	0.9150

Supplementary Table 4. HER performance comparisons The comparison of the C-$Ni_{1-x}O$/NF with the reported state-of-the-art nickel-based sulfides, nitrides, and phosphides.

Catalyst	η (mV) at j_{geo}=10 mA cm^{-2}	Scan rate (mV s^{-1})	Tafel slope (mV dec^{-1})	Reference
**C-$Ni_{1-x}O$/NF**	**27**	**1**	**36**	**This work**
NiS_2 Hollow microsphere	219	5	157	5
Cu nanodot decorated Ni_3S_2 nanotubes	128	5	76.2	6
Activated NiS_2 nanosheets /NF	67	1	63	7
Ni-N_x/porous carbon	147	1	114	8
Ni_3N_{1-x}/NF	55	5	54	9
Ni_3N nanosheets/Pt	50	5	36.5	10
Ni_2P nanoparticles/NF	221	5	-	11
Ni_5P_4 film/NF	150	10	53	12
Ni_2P flakes/NF	85	5	-	13

Supplementary Table 5. The summary of total energy $\boldsymbol{E}$, solvation energy $\boldsymbol{E_{solvation}}$, vibrational energy $\boldsymbol{E_{vibraion}}$ and free energy $\boldsymbol{G}$ for different surfaces. For total energy $\boldsymbol{E}$, o-surface and C-surface are using their corresponding initial state (IS) as reference point. For $\boldsymbol{G'}$, o-surface and C-surface are using their corresponding IS free energy $\boldsymbol{G}$ as reference point

Surface	$\boldsymbol{E}$ $(\boldsymbol{eV})$	$\boldsymbol{E_{solvation}}(\boldsymbol{eV})$	$\boldsymbol{E_{vibraion}}$ $(\boldsymbol{eV})$	$\boldsymbol{G}$ $(\boldsymbol{eV})$	$\boldsymbol{G'}$ $(\boldsymbol{eV})$
IS of o-surface	0	-0.598	1.270	0.672	0
TS of o-surface	1.23	-0.508	1.117	1.809	1.167
FS of o-surface	1.04	-0.545	1.036	1.531	0.859
IS of C-surface	0	-0.600	1.276	0.676	0
TS of C-surface	0.8	-0.549	1.232	1.483	0.807
FS of C-surface	0.65	-0.516	1.262	1.396	0.720

Supplementary Notes

Supplementary Note 1

Formation of nickel oxides

The dehydration of nickel oxalate dihydrate can result in nickel oxalate and water ($NiC_2O_4 \cdot 2H_2O \rightarrow NiC_2O_4 + 2H_2O$) and the subsequent decomposition of nickel oxalate leads to the generation of metallic Ni and carbon dioxide ($NiC_2O_4 \rightarrow Ni + 2CO_2$). During the rapid decomposition of nickel oxalate, the carbon dopants can be retained in the Ni structure. Furthermore, the residual water from the dehydration step can react with Ni at the decomposition temperature ($2H_2O + Ni \rightarrow NiO + H_2$), leading to the formation of nickel oxides[14,15].

Supplementary Note 2

Determination of stable surface

To determine the most stable surface termination, a surface phase diagram was plotted as a function of chemical potential of O species. In general, the surface energy is defined as the energy required to form new surface divided by the surface area.

$$\gamma = \frac{1}{A}\left(E_{slab}^{relaxed} - E_{fixed\,part} - \mu_{Ni}N_{Ni} - \mu_O N_O\right) \quad (1)$$

where, $E_{slab}^{relaxed}$ is the energy for the whole relaxed slab, $E_{fixed\,part}$ is the energy for the fixed bottom two layers, μ_{Ni} and μ_O are the chemical potential of nickel and oxygen, respectively, and N_{Ni} and N_O are the number of nickel and oxygen ions in the relaxed part. For NiO, the following chemical potential relation of Ni and O must be satisfied.

$$\mu_{Ni} + \mu_O = \mu_{NiO} \quad (2)$$

Since there are 8 Ni and 8 O in NiO bulk unit cell, we have

$$\mu_{NiO} = \frac{1}{8}E_{NiO\,bulk}. \quad (3)$$

Enforcing equation (1), (2) and (3) has allowed us to now write the surface energy as a function of μ_O

$$\gamma = \frac{1}{A}\left[E_{slab}^{relaxed} - E_{fixed\,part} - \frac{1}{8}E_{NiO\,bulk}N_{Ni} + \mu_O(N_{Ni} - N_O)\right] \quad (4)$$

Finally, we look to determine the range of μ_O. In an oxygen rich environment, oxygen will come from O_2 gas and hence $\mu_O = \frac{1}{2}E_{O_2}$. In a nickel rich environment (oxygen poor), nickel will be produced from pure bulk Ni and so $\mu_O = \frac{1}{8}E_{NiO} - E_{Ni}$. These extreme limits give upper and lower bounds for μ_O.

$$\frac{1}{8}E_{NiO} - E_{Ni} \leq \mu_O \leq \frac{1}{2}E_{O_2} \quad (5)$$

According to equation (4), we plotted how surface energy changes with μ_O in the range shown in equation (5) in Supplementary Fig. 6.

Supplementary Note 3

Chemical environment of C-Ni_{1-x}O-Air

In order to investigate the role of C in HER, C-Ni_{1-x}O was annealed in the air at 400 °C for 10 min (denoted as C-Ni_{1-x}O-Air) to remove the C dopant. SEM images (Supplementary Fig. 12) show that C-Ni_{1-x}O-Air exhibits the same morphological feature with that of C-Ni_{1-x}O after the post-growth air annealing. The XPS data (Supplementary Fig. 13) collected from C-Ni_{1-x}O-Air suggest that the carbon dopants in C-Ni_{1-x}O have been removed after the air annealing, which is evidenced by the disappearance of the C dopant signal (288.6 eV in C1s spectrum and 855.7 eV in Ni 20 spectrum). The Ni 2p and O 1s spectra suggests the presence of NiO. Specifically, the peaks at 853.3 eV in Ni 2p and 529 eV in O 1s spectra suggests the existence of NiO[16,17], while the peak at 855.2 eV originates from Ni^{3+} in NiO[18,19]. The presence of Ni^{3+} is a result of the formation of Ni vacancies (V_{Ni}) in NiO, as evidenced by O-V_{Ni} at 530.9 eV in O 1s spectra[20,21]. The 532.7 eV signal in O 1s spectra is attributed to the surface adsorbed OH on the NiO[22], while the peak centered at 532 eV together with the other two peaks (286.2 and 288.1 eV) in C 1s spectra are corresponding to the adsorbed oxidized adventitious carbon[16,23]. Taking together, SEM and XPS results support that C-Ni_{1-x}O-Air can serve as a control sample to investigate the effect of carbon doping.

Supplementary Note 4

Chemical environment of C-$Ni_{1-x}O$ after CV conditioning

By comparing the XPS results (Supplementary Fig. 14) with those obtained from the C-$Ni_{1-x}O$ catalyst before conditioning (Fig. 3), the peaks located at 854.5, 855.7 and 857.1 eV in Ni 2p spectrum corresponding to Ni^{2+} in NiO, Ni-O-C=O, and Ni^{3+} are all present for the conditioned sample (Supplementary Fig. 14a), suggesting the oxidative Ni species and carbon dopant are preserved. Likewise, the characteristic peaks at 528.5, 529.4, 530.9 eV, and 532.3 eV in O 1s spectra, and the peaks at 284.6 and 288.6 eV in C 1s spectra are also consistent with the signals obtained from the catalyst before conditioning (Fig. 3a). The O and C XPS results also confirm that the existence of the Ni^{2+}, Ni vacancy, and the dopant structure of O-C=O after conditioning. The extra peaks (533 eV in O 1s, 286.8, 289.9, and 291.8 eV in C 1s spectra) observed for the conditioned sample are ascribed to the C-O and C-F/C-F_2 structure from the residues of Nafion binder that was used in attaching the catalyst to the electrode[24-27]. The Nafion residues in the conditioned sample can also result in a C 1s peak at 288.6 eV due to its C-F_2S structure[25]. This peak overlaps with that of O-C=O, which makes it difficult to estimate the atomic carbon concentration after conditioning. In order to exclude the influence of Nafion binder and confirm the coordination environment, XANES characterizations were carried out on the binder-free catalyst sample, i.e. C-$Ni_{1-x}O$ grown on nickel foam (Supplementary Fig. 14b). The highlighted shaded areas of O K-edge and C K-edge spectra show the exact same characteristic peaks of C-$Ni_{1-x}O$ reported in Fig. 3. The results confirm that the O-C=O structure (carbon doping) are still presented in the conditioned sample. Taken together the XPS and XANES results, it is reasonable to conclude that the catalyst's composition is similar before and after conditioning. Furthermore, based on our XPS and XANES, it is reasonable to conclude that NiO layer is preserved after measurement.

Supplementary Methods

DFT Calculation Details

For the QE calculation part, ultrasoft pseudopotential with kinetic energy cutoffs of 40 Ry for wavefunction and 240 Ry for charge density is implemented. In order to capture the correct antiferromagnetic ordering of Ni along the (111) direction, a $2 \times 2 \times 2$ supercell must be used. Integration over the Brillouin zone is performed using a $4 \times 4 \times 4$ k-point mesh. In order to improve k-point integration, a cold smearing of 0.002 Ry is used[28]. A convergence threshold of $1.0E^{-7}$ Ry on total energy is used for all self-consistency calculations. Atomic positions are relaxed until the total energy and total force converges to $1.0E^{-4}$ Ry and $1.0E^{-3}$ Ry $Bohr^{-1}$, respectively. The calculated lattice constant, magnetic moment and band gap are 4.22 Å, 1.64 μ_B and 2.74 eV, which agree well with previously reported theoretical values of 4.19 Å, 1.69 μ_B and 2.43 eV[2,4]. These values are listed in Supplementary Table 1.

In order to study the surface of NiO, the bulk unit cell was converted from its fcc unit cell to simple cubic unit cell, and then this cell was expanded to a $\sqrt{5} \times \sqrt{2} \times 1$ supercell for the (111) surface or a $2 \times 2 \times 1$ supercell for the (100) surface. A vacuum region of 15 Å is added to effectively separate two adjacent slabs and avoid spurious interactions. The convergence of surface energy over the number of layers was tested and it was found that the energy difference between 5-layer slab and 7-layer slab is smaller than 1 meV $Å^{-2}$, so 5-layer slab is used. In order to mimic the properties of bulk, the bottom two layers of (111) surface were fixed with bulk positions, while the remaining structure relaxed as the surface. Due to the inversion symmetry breaking, a dipole field correction was applied along the vacuum direction. For all slab calculations, a $2 \times 2 \times 1$ k-point mesh was used with a smearing of 0.005 Ry to help the convergence.

Calculation of ΔG_H

ΔG_H is the most commonly used indicator to compare the activity between different sites toward hydrogen adsorption since it correlates well with experimental exchange current densities, and an optimal hydrogen adsorption corresponds to $\Delta G_H = 0$ (too positive or negative ΔG_H leads to too weak or strong hydrogen adsorption)[29]. We calculated ΔG_H with the following equation, proposed by Nørskov et al[30,31].

$$\Delta G_H = \Delta E_H + T\Delta S + \Delta ZPE + \Delta E_{solvation} \qquad (6)$$

where, ΔE_H is the change in the total energy change after H adsorbs on the surface, $T\Delta S$ is the change of entropy, ΔZPE is the change of zero point energy and $\Delta E_{solvation}$ is the solvation energy difference between surface with H and bare surface.

H adsorption profile on p-surface and o-surface

The ΔG_H of all possible hydrogen adsorption sites on both surfaces have been calculated to give insights into the effect of Ni vacancy on the activity toward hydrogen adsorption. With respect to p-surface, we found that only the bridge site (i.e. H bonding with two Ni, Supplementary Fig. 7) is favorable for H adsorption with a ΔG_H value of -0.192 eV (Supplementary Table 2). On the other hand, for the o-surface, in addition to Ni #1 (Supplementary Fig. 8a), both the exposed third layer Ni and second layer O are also possible H adsorption sites. The ΔG_H of Ni #1 exhibits a more positive value of 0.935 eV as expected as the formation of Ni vacancy ($Ni^{2+} = V_{Ni} + 2h^{+}$) is accompanied with oxidization of the nearby Ni^{2+} to Ni^{3+}, making H adsorption more difficult on Ni #1. In contrast, the exposed underlayer Ni and O sites of the o-surface are more favorable for H adsorption. The hollow sites composed by three-fold third layer Ni ions (Supplementary Fig. 8b) give a slightly positive ΔG_H of 0.152 eV (Supplementary Table 2) because the three-bonds configuration largely increases the interaction between Ni and H. The second layer O sites (Supplementary Fig. 8c) exhibit a most close to 0 value of $\Delta G_H = -0.098$ eV. However, we found that the adsorption of H_2 molecules generated in HER onto the second layer O ions are too favorable that the subsequent relax process of H_2 would cause one H strongly bonds to the O site and the other one leaves. This result suggests that the O sites could be occupied/deactivated by H_2 molecules during HER. Similar situation was not found on the Ni sites which are expected to be active during HER. Overall, o-surface doesn't increase the number of active H adsorption sites per surface area compared to that of p-surface.

Calculation of ECSA

The ECSA was calculated based on a method reported in literature[32]. A series of CV (from 20 to 120 mV s^{-1} with an interval of 20 mV s^{-1}) were collected in a non-faradaic reaction potential window from 0.2 V to 0.1 V vs. RHE. A linear plot of the j_{anodic} - $j_{cathodic}$ versus scan rate was

obtained accordingly and the slope is proportional to the ECSA. The ECSA can be calculated through the following equation:

$$ECSA = \frac{C_{areal} \times A}{C_{ref}} \qquad (7)$$

Where C_{areal} represents the areal capacitance (i.e. the slope of Supplementary Fig. 14), A is the geometric area of the working electrode (0.5 cm^2), and C_{ref} is the referential area capacitance of flat electrode (here we use 40 μF cm^{-2} for Ni based catalysts and 30 μF cm^{-2} for Pt suggested by Jaramillo et al.[33,34]).

Energy barrier calculation

For the energy barrier calculation, the IS and FS for Heyrovsky step on both o-surface and C-surface were relaxed. The number of required IMs for NEB calculations was tested for the Heyrovsky reaction over o-surface and C-surface. It was found that 8 IMs and 6 IMs are enough to converge the barriers for o-surface and C-surface, respectively. After the exact saddle point was found by DIMER calculation, we did phonon calculation for all atoms in the system (except for the layers whose atoms are fixed) to check the number of imaginary frequencies in the system and confirmed there is only one imaginary frequency, whose direction is along the reaction pathway. All these indicate that they are the real saddle points.

Coordinates files

The coordinates files for pristine NiO surface, octopolar NiO surface and C doped NiO surface (the atoms with "0 0 0" after their coordinates are fixed at the NiO bulk positions, in order to recover the bulk properties at the bottom layer of the slab) are shown below.

Pristine NiO:

```
ATOMIC_POSITIONS (angstrom)
Ni2     2.585001230   1.492451429   0.000000000    0   0   0
Ni2    12.925001144   7.462253571   0.000000000    0   0   0
Ni2     7.755000591   4.477352142   0.000000000    0   0   0
Ni2     7.755013943   7.462252617   0.000000000    0   0   0
Ni2     7.754985332   1.492450356   0.000000000    0   0   0
Ni2     2.585014105   4.477351665   0.000000000    0   0   0
Ni2    10.339985847   2.984901428   0.000000000    0   0   0
Ni2     5.170014381   5.969802380   0.000000000    0   0   0
Ni2    10.340000153   5.969802380   0.000000000    0   0   0
Ni2     5.170000076   2.984901190   0.000000000    0   0   0
Ni2     0.000000000   0.000000000   0.000000000    0   0   0
```

Ni2	5.169985771	0.000000000	0.000000000	0	0	0
Ni2	6.634893426	5.949060775	4.899562956			
Ni2	11.803075597	2.977797710	4.899414562			
Ni2	11.789835516	8.956480205	4.913410004			
Ni2	12.100744959	5.966058055	4.820391291			
Ni2	1.769263106	0.007260560	4.816415868			
Ni2	6.925002264	3.001664437	4.815725509			
Ni2	4.388141356	1.274623526	4.878680099			
Ni2	4.402543623	7.230907084	4.886677723			
Ni2	9.572910959	4.259264754	4.879407637			
Ni2	9.565357497	7.672709186	4.880994308			
Ni2	9.579401780	1.702201992	4.876635407			
Ni2	4.406890533	4.674057545	4.872048574			
O	9.478335381	7.462243557	1.217316151	0	0	0
O	4.308336735	4.477342129	1.217316151	0	0	0
O	9.478307724	1.492441773	1.217316151	0	0	0
O	1.723326445	-0.000005241	1.217316151	0	0	0
O	12.063324928	5.969794273	1.217316151	0	0	0
O	6.893324852	2.984893084	1.217316151	0	0	0
O	4.308324337	1.492440701	1.217317343	0	0	0
O	4.308351040	7.462243080	1.217317343	0	0	0
O	9.478323936	4.477341652	1.217317343	0	0	0
O	1.723338723	2.984895229	1.217317343	0	0	0
O	6.893338680	5.969796181	1.217317343	0	0	0
O	6.893310547	-0.000005241	1.217317343	0	0	0
O	7.766789645	4.497327788	3.760836568			
O	2.593344587	1.518328043	3.762554610			
O	12.932300029	7.479346140	3.771802485			
O	5.169473594	5.966141917	3.430139259			
O	10.332160741	2.974859228	3.425497528			
O	5.166486136	-0.001256182	3.432949109			
O	7.759884848	7.435675829	3.774912341			
O	2.597434813	4.454770581	3.774813658			
O	7.764210268	1.465795600	3.797857620			
O	5.156336003	2.974137518	3.821763842			
O	10.330291347	5.972079800	3.830678052			
O	-0.014826790	-0.008754839	3.846753535			
Ni1	6.041919473	4.464512861	2.388429385			
Ni1	11.216181937	1.480003769	2.393095547			
Ni1	11.210076728	7.440808018	2.423753153			
Ni1	6.050819639	7.471160410	2.407281049			
Ni1	11.211025812	4.479782930	2.400897050			
Ni1	6.039044278	1.511145077	2.411448217			
Ni1	3.446911931	2.987146136	2.474052860			
Ni1	8.616437798	5.965441024	2.485672225			
Ni1	13.794849099	8.956191981	2.516308698			

Ni1 8.597749012 8.958600837 2.394043722
Ni1 8.597931860 2.987027572 2.388740778
Ni1 13.771277194 5.962818379 2.403863855

Octopolar NiO (Ni terminated):

ATOMIC_POSITIONS (angstrom)

Ni2	2.585001230	1.492451429	0.000000000	0	0	0
Ni2	12.924998283	7.462252617	0.000000000	0	0	0
Ni2	7.754999638	4.477351665	0.000000000	0	0	0
Ni2	7.755012512	7.462250710	0.000000000	0	0	0
Ni2	2.585012436	4.477349758	0.000000000	0	0	0
Ni2	7.754984856	1.492449880	0.000000000	0	0	0
Ni2	5.170012474	5.969799995	0.000000000	0	0	0
Ni2	10.339982986	2.984899759	0.000000000	0	0	0
Ni2	5.169984818	0.000000000	0.000000000	0	0	0
Ni2	0.000000000	0.000000000	0.000000000	0	0	0
Ni2	10.339996338	5.969800949	0.000000000	0	0	0
Ni2	5.169998169	2.984900475	0.000000000	0	0	0
Ni2	4.306194243	1.501121326	4.551515661			
Ni2	14.653750778	7.466375769	4.551909056			
Ni2	9.475588783	4.475231805	4.544669233			
O	9.478334427	7.462242126	1.217316151	0	0	0
O	4.308336258	4.477341652	1.217316151	0	0	0
O	9.478305817	1.492441177	1.217316151	0	0	0
O	1.723326445	-0.000005522	1.217316151	0	0	0
O	12.063322067	5.969792843	1.217316151	0	0	0
O	6.893323421	2.984893322	1.217316151	0	0	0
O	4.308324337	1.492441058	1.217317343	0	0	0
O	4.308349133	7.462241650	1.217317343	0	0	0
O	9.478322983	4.477341175	1.217317343	0	0	0
O	6.893336773	5.969793797	1.217317343	0	0	0
O	1.723337412	2.984893322	1.217317343	0	0	0
O	6.893309593	-0.000005522	1.217317343	0	0	0
O	5.123546743	2.930225968	3.578302127			
O	5.145779187	8.887183318	3.578899865			
O	10.303952888	5.907218999	3.584039903			
O	7.830262748	4.472456026	3.568565900			
O	2.658753682	1.488053427	3.578888954			
O	13.004298309	7.472723515	3.583152348			
O	5.128409066	6.033814232	3.577497797			
O	10.296843667	3.049217762	3.566944854			
O	5.138734648	0.076295147	3.581518558			
Ni1	8.638884009	5.933051406	2.402611468			
Ni1	3.461230128	2.953235312	2.405000067			
Ni1	13.814659285	8.927158160	2.409689826			
Ni1	5.995361486	7.459025161	2.404989384			

```
Ni1     11.156261065   4.477428660   2.401255993
Ni1      5.992329088   1.502653856   2.408652273
Ni1      3.467857197   0.032478394   2.411450932
Ni1      3.464927250   6.003800658   2.411637290
Ni1      8.638380494   3.021776336   2.381785149
Ni1      6.032602829   4.478666580   2.681399014
Ni1     11.200626483   1.494791358   2.680944137
Ni1     11.202390257   7.465695549   2.707438063
```

C doped NiO:

```
ATOMIC_POSITIONS (angstrom)
Ni2      3.953368411   8.105519390   4.769924411
Ni2      3.919601218   0.851430979   4.769428920
Ni2     10.228399643   4.464283009   4.768965457
Ni2      2.585001230   1.492451549   0.000000000    0   0   0
Ni2     12.924998283   7.462253094   0.000000000    0   0   0
Ni2      7.755012035   7.462251663   0.000000000    0   0   0
Ni2      7.754999638   4.477351665   0.000000000    0   0   0
Ni2      5.170012474   5.969800949   0.000000000    0   0   0
Ni2      7.754984856   1.492450118   0.000000000    0   0   0
Ni2      2.585011959   4.477350235   0.000000000    0   0   0
Ni2     10.339996338   5.969801426   0.000000000    0   0   0
Ni2      5.169998169   2.984900713   0.000000000    0   0   0
Ni2     10.339982033   2.984900236   0.000000000    0   0   0
Ni2      5.169984818   0.000000000   0.000000000    0   0   0
Ni2      0.000000000   0.000000000   0.000000000    0   0   0
O       10.166559505   5.947766858   3.548773144
O       13.032175701   7.320918067   3.549691981
O        5.234497600   0.161388833   3.549429723
O        5.248501762   8.793523110   3.528300056
O        2.682636983   1.638608160   3.529576713
O        5.379463072   3.345821320   3.523053475
O        5.381944586   5.605949389   3.524350303
O        7.337672278   4.473901882   3.522837971
O       10.164907344   2.997895871   3.529188974
O        9.478322983   4.477342129   1.217317343    0   0   0
O        4.308348179   7.462242603   1.217317343    0   0   0
O        4.308324337   1.492441177   1.217317343    0   0   0
O        6.893309593  -0.000005802   1.217317343    0   0   0
O        1.723336935   2.984893560   1.217317343    0   0   0
O        6.893337250   5.969794750   1.217317343    0   0   0
O        9.478334427   7.462243080   1.217316151    0   0   0
O        4.308336258   4.477342606   1.217316151    0   0   0
O        9.478305817   1.492441177   1.217316151    0   0   0
O        6.893323898   2.984893560   1.217316151    0   0   0
O        1.723326206  -0.000005802   1.217316151    0   0   0
```

O	12.063321114	5.969794273	1.217316151	0	0	0
Ni1	11.202545510	7.462588279	2.735532173			
Ni1	11.201144509	1.491540669	2.691088910			
Ni1	13.877250805	5.939230694	2.439927652			
Ni1	3.488650717	0.079140634	2.419721085			
Ni1	13.834048077	8.876090325	2.421822169			
Ni1	6.013095990	1.583746641	2.439877144			
Ni1	3.525379428	3.020093177	2.420554667			
Ni1	6.023027430	7.373537820	2.419918484			
Ni1	8.546335249	5.906985532	2.439458465			
Ni1	11.112319135	4.476004809	2.418688105			
Ni1	8.546482919	3.035853130	2.420442764			
C	6.033012093	4.475103469	3.498717881			

Supplementary Reference

1. Sawatzky, G. A. & Allen, J. W. Magnitude and origin of the band gap in NiO. *Phys*. *Rev*. *Lett*. **53**, 2339-2342 (1984).
2. Zhang, W. & Tang, B. Stability of the polar NiO (111) surface. *J. Chem*. *Phys*. **128**, 124703 (2008).
3. Cheetham, A. K. & Hope, D. A. O. Magnetic ordering and exchange effects in the antiferromagnetic solid solutions $Mn_xNi_{1-x}O$. *Phy*. *Rev*. *B* **27**, 6964-6967 (1983).
4. Li, L. & Kanai, Y. Antiferromagnetic structures and electronic energy levels at reconstructed NiO (111) surfaces: A DFT + U study. *Phy*. *Rev*. *B* **91**, 235304 (2015).
5. Tian ,T., Huang, L., Ai, L. & Jiang, J. Surface anion-rich NiS_2 hollow microspheres derived from metal–organic frameworks as a robust electrocatalyst for the hydrogen evolution reaction. *J. Mater*. *Chem*. *A* **5**, 20985-20992 (2017).
6. Feng, J., Wu, J., Tong, Y. & Li, G. Efficient hydrogen evolution on Cu nanodots-decorated Ni_3S_2 nanotubes by optimizing atomic hydrogen adsorption and desorption. *J*. *Am*. *Chem*. *Soc*. **140**, 610-617 (2018).
7. Ma, Q. *et al.* Identifying the electrocatalytic sites of nickel disulfide in alkaline hydrogen evolution reaction. *Nano Energy* **41**, 148-153 (2017).
8. Lei, C. *et al.* Efficient alkaline hydrogen evolution on atomically dispersed Ni–N_x Species anchored porous carbon with embedded Ni nanoparticles by accelerating water dissociation kinetics. *Energy Environ*. *Sci*. **12**, 149-156 (2019).
9. Liu, B. *et al.* Unconventional nickel nitride enriched with nitrogen vacancies as a high-efficiency electrocatalyst for hydrogen evolution. *Adv*. *Sci*. **5**, 1800406 (2018).
10. Wang, Y., Chen, L., Yu, X., Wang, Y. & Zheng, G. Superb alkaline hydrogen evolution and simultaneous electricity generation by Pt-decorated Ni_3N nanosheets. *Adv*. *Energy Mater*. **7**, 1601390 (2017).
11. Stern, L. A., Feng, L., Song, F. & Hu, X. Ni_2P as a Janus catalyst for water splitting: the oxygen evolution activity of Ni_2P nanoparticles. *Energy Environ*. *Sci*. **8**, 2347-2351 (2015).
12. Ledendecker, M. *et al.* The synthesis of nanostructured Ni_5P_4 films and their use as a non-noble bifunctional electrocatalyst for full water splitting. *Angew*. *Chem*. *Int*. *Ed*. **54**, 12361-12365 (2015).

13. Menezes, P. W. *et al.* Uncovering the nature of active species of nickel phosphide catalysts in high-performance electrochemical overall water splitting. *ACS Catal*. **7**, 103-109 (2017).
14. Carney, C. S., Gump, C. J. & Weimer, A. W. Rapid nickel oxalate thermal decomposition for producing fine porous nickel metal powders: Part 1: Synthesis. *Mater. Sci. Eng. A* **431**, 1-12 (2006)
15. Mansour, S. A. A. Spectroscopic and microscopic investigations of the thermal decomposition course of nickel oxysalts: Part 3. Nickel oxalate dihydrate. *Thermochim. Acta* **230**, 243-257 (1993).
16. Dalavi, D. S., Devan, R. S., Patil, R. S., Ma, Y.-R. & Patil, P. S. Electrochromic performance of sol–gel deposited NiO thin film. *Mater. Lett*. **90**, 60-63 (2013).
17. Cai, G. F. *et al.* An efficient route to a porous NiO/reduced graphene oxide hybrid film with highly improved electrochromic properties. *Nanoscale* **4**, 5724-5730 (2012).
18. Kim, D., Ryu, J., Manders, J., Lee, J. & So, F. Air-stable, solution-processed oxide p–n heterojunction ultraviolet photodetector. *ACS Appl. Mater. Interfaces* **6**, 1370-1374 (2014).
19. Gomaa, M. M. *et al.* Exploring NiO nanosize structures for ammonia sensing. *J. Mater. Sci.: Mater. Electron*. **29**, 11870-11877 (2018).
20. Tomellini, M. X-ray photoelectron spectra of defective nickel oxide. *J. Chem. Soc., Faraday Trans. 1* **84**, 3501-3510 (1988).
21. Sasi, B. & Gopchandran, K. G. Nanostructured mesoporous nickel oxide thin films. *Nanotechnology* **18**, 115613 (2007).
22. Jang. W.-L., Lu, Y.-M. & Hwang, W.-S. Effect of different atmospheres on the electrical stabilization of NiO films. *Vacuum* **83**, 596-598 (2008).
23. Mansour, A. N. Characterization of NiO by XPS. *Surf. Sci. Spectra* **3**, 231-238 (1994).
24. Su, W., Li, Z., Liu, S. & Ding, X. Indirect electrochemical detection of NADH through an active stainless steel fiber felt (SSFF) electrode decorated with the amino-graphene/nafion nano composite films. *ChemistrySelect* **3**, 6214-6220 (2018).
25. Li, H.-Y & Liu, Y.-L. Nafion-functionalized electrospun poly(vinylidene fluoride) (PVDF) nanofibers for high performance proton exchange membranes in fuel cells. *J. Mater. Chem. A* **2**, 3783-3793 (2014).
26. Chen, C., Levitin, G., Hess, D. W. & Fuller, T. F. XPS investigation of nafion® membrane degradation. *J. Power Sources* **169**, 288-295 (2007).

27. Xu, J., Zhang, Q. & Cheng, Y.-T. High capacity silicon electrodes with nafion as binders for lithium-ion batteries. *J. Electrochem. Soc.* **163**, A401-A405 (2016).

28. Marzari, N., Vanderbilt, D., De Vita, A. & Payne, M. C. Thermal contraction and disordering of the Al (110) surface. *Phys. Rev. Lett.* **82**, 3296-3299 (1999).

29. Greeley, J., Jaramillo, T. F., Bonde, J., Chorkendorff, I. & Nørskov, J. K. Computational high-throughput screening of electrocatalytic materials for hydrogen evolution. *Nat. Mater.* **5**, 909 (2006).

30. Nørskov, J. K. *et al*. Trends in the exchange current for hydrogen evolution. *J. Electrochem. Soc.* **152**, J23-J26 (2005).

31. Ping, Y., Nielsen, R. J. & Goddard, W. A. The reaction mechanism with free energy barriers at constant potentials for the oxygen evolution reaction at the IrO_2 (110) surface. *J. Am. Chem. Soc.* **139**, 149-155 (2017).

32. Hu, C. *et al*. In situ electrochemical production of ultrathin nickel nanosheets for hydrogen evolution electrocatalysis. *Chem* **3**, 122-133 (2017).

33. McCrory, C. C. L. et al. Benchmarking hydrogen evolving reaction and oxygen evolving reaction electrocatalysts for solar water splitting devices. *J. Am. Chem. Soc.* **137**, 4347-4357 (2015).

34. McCrory, C. C. L., Jung, S., Peters, J. C. & Jaramillo, T. F. Benchmarking heterogeneous electrocatalysts for the oxygen evolution reaction. *J. Am. Chem. Soc.* **135**, 16977-16987 (2013).
